# Dynamic Regulation of Cell Volume and Extracellular ATP of Human Erythrocytes

**DOI:** 10.1371/journal.pone.0158305

**Published:** 2016-06-29

**Authors:** M. Florencia Leal Denis, H. Ariel Alvarez, Natalia Lauri, Cora L. Alvarez, Osvaldo Chara, Pablo J. Schwarzbaum

**Affiliations:** 1 Instituto de Química y Fisicoquímica Biológicas “Prof. A. C. Paladini”, Universidad de Buenos Aires, CONICET, FFyB, Buenos Aires, Argentina; 2 Instituto de Física de Líquidos y Sistemas Biológicos (IFLYSIB), CONICET, Universidad Nacional de La Plata (UNLP), La Plata, Argentina; 3 Center for Information Services and High Performance Computing (ZIH), Technische Universität Dresden (TUD), Dresden, Germany; Albany Medical College, UNITED STATES

## Abstract

**Introduction:**

The peptide mastoparan 7 (MST7) triggered in human erythrocytes (rbcs) the release of ATP and swelling. Since swelling is a well-known inducer of ATP release, and extracellular (ATPe), interacting with P (purinergic) receptors, can affect cell volume (Vr), we explored the dynamic regulation between Vr and ATPe.

**Methods and Treatments:**

We made a quantitative assessment of MST7-dependent kinetics of Vr and of [ATPe], both in the absence and presence of blockers of ATP efflux, swelling and P receptors.

**Results:**

In rbcs 10 μM MST7 promoted acute, strongly correlated changes in [ATPe] and Vr. Whereas MST7 induced increases of 10% in Vr and 190 nM in [ATPe], blocking swelling in a hyperosmotic medium + MST7 reduced [ATPe] by 40%. Pre-incubation of rbcs with 10 μM of either carbenoxolone or probenecid, two inhibitors of the ATP conduit pannexin 1, reduced [ATPe] by 40–50% and swelling by 40–60%, while in the presence of 80 U/mL apyrase, an ATPe scavenger, cell swelling was prevented. While exposure to 10 μM NF110, a blocker of ATP-P2X receptors mediating sodium influx, reduced [ATPe] by 48%, and swelling by 80%, incubation of cells in sodium free medium reduced swelling by 92%.

**Analysis and Discussion:**

Results were analyzed by means of a mathematical model where ATPe kinetics and Vr kinetics were mutually regulated. Model dependent fit to experimental data showed that, upon MST7 exposure, ATP efflux required a fast 1960-fold increase of ATP permeability, mediated by two kinetically different conduits, both of which were activated by swelling and inactivated by time. Both experimental and theoretical results suggest that, following MST7 exposure, ATP is released via two conduits, one of which is mediated by pannexin 1. The accumulated ATPe activates P2X receptors, followed by sodium influx, resulting in cell swelling, which in turn further activates ATP release. Thus swelling and P2X receptors constitute essential components of a positive feedback loop underlying ATP-induced ATP release of rbcs.

## Introduction

Human erythrocytes (rbcs) release ATP following exposure to hypoxia, β-adrenergic stimulation, prostacyclin analogs, acidity and/or mechanical stress [1,2].

These treatments mimic the physiological conditions to which rbcs are exposed in the vasculature, e.g. when passing through the small branches of the microcirculation [3–7].

A signaling route promoting ATP release in rbcs involves dissociation of heterotrimeric Gi, allowing βγ dimers to stimulate specific isoforms of adenylyl cyclases, and the subsequent increase in cAMP formation. These events are followed by a series of not-well defined intracellular signaling events upstream of ATP release [8–10]. In mammalian rbcs, this route was proposed to transduce mechanical stress and hypoxia into ATP release [11,12]. Similarly, the tetradecapeptide mastoparan binds to the plasma membrane and forms an amphiphilic helix that activates Gi without requiring the activation of a receptor [13]. Its potent analog mastoparan 7 (MST7), synthesized by substituting alanine for the positively charged lysine in position 12, acts as a potent analog of MST in rbcs and other cell systems [10,14]. Several reports show that MST and MST7 activate regulated ATP efflux of rbcs [10].

Once in the extracellular medium, extracellular ATP (ATPe) can trigger different cellular responses by interacting with P receptors (purinergic receptors, [15,16] on the cell surface while at the same time its concentration is controlled by the activities of one or more ectonucleotidases [17].

*In vivo*, the physiological effects of such ligand-receptor interactions can be manifold. For example, ATP release of mammalian rbcs was shown to control the distribution of perfusion in the microvasculature of skeletal muscle [4,6]. In the vascular lumen, rbc-derived ATP produced not only local vasodilation, but also dilation that was conducted to upstream arterioles [1,18].

*In vitro*, an hypotonic treatment inducing swelling constitutes a potent stimulus of ATP release in most cell types. Swelling and ATP release appear closely intertwined due to common, sometimes overlapping factors affecting them [19].

Swelling can alter the permeability of diffusible ions by activating specific channels, some of which were postulated to facilitate ATP exit [20–22]. ATPe, on the other hand, can activate ionotropic P receptors (P2X) that mediate the net influx of Na^+^ and/or Ca^++^ [23–25], thereby affecting intracellular osmolarity and ultimately cell volume.

In hypotonic conditions, the relationship between ATPe (and byproducts) and cell volume has been almost exclusively studied in the context of a volumetric response termed volume regulatory decrease or RVD, which is present in most animal cells. Several reports showed that ATPe, and or its metabolic products generated by ectonucleotidase activity, interact with P receptors to mediate RVD [26–29]. It then became clear that, not only swelling could activate ATP efflux, but that the subsequent increase of [ATPe] could active P receptors and induce RVD.

However, in rbcs, as well as in erythrocytes of most mammalian species, the situation is different, since these cells lack volume regulatory mechanisms [30,31] or exhibited a very limited capacity for volume regulation [32]. That is, erythrocytes can swell or shrink as dictated only by their osmotic environment. Still, rbcs can swell under hypotonicity imposed by either a decrease of extracellular osmolarity, or an increase of intracellular osmolarity, a condition where ATP release was verified [29,32].

Rbcs lack intracellular compartments, so that no exocytotic ATP release can occur. Candidate conduits for ATP release include channels such as the maxi anion channel, volume regulated ion channel and tweety, pore forming proteins like connexins, pannexins and P2X7 receptors [33].

Activation of one or more ATP release pathways depends on the nature of the stimulus [2,29]. Under a robust hypotonic treatment rbcs were able to release ATP primarily through pannexin 1 [34], whereas mice rbcs responded to a hypotonic- depolarizing stimulus by triggering two different ATP release pathways [35]. In murine fibrosarcoma cells, on the other hand, maxi anion channels and pannexin 1 were shown to constitute separate permeation pathways for swelling induced ATP release [36].

In this study we seek to explore the relationship between cell volume and ATP release of rbcs. For this purpose cells were exposed to the peptide mastoparan 7 (MST7).

We made a quantitative assessment of the kinetics of cell volume and [ATPe] under similar experimental conditions, both in the absence and presence of blockers of ATP efflux, swelling and P receptors.

The dynamic regulation of cell volume and ATPe was further analyzed by means of a data-driven mathematical model. Model dependent fit to experimental data showed that ATP release is mediated by two kinetically different ATP conduits, both of which were affected by cell swelling.

Both experimental and theoretical results suggest that cell swelling and P2X receptors are essential components of a positive feedback loop underlying ATP-induced ATP release of rbcs.

## Materials and Methods

### Chemicals

All reagents in this study were of analytical grade. Poly-D-lysine, mastoparan7, bovine serum albumin, carbenoxolone, probenecid, firefly luciferase (EC 1.13.12.7), choline chloride, suramin, PPADS, NF110, imidazole, sucrose, Hepes, apyrase, POM1, 2-MeSADP, MRS 2211 and HIBA were purchased from Sigma-Aldrich (St. Louis, MO, USA). BCECF-AM and D-luciferin were purchased from Invitrogen/Molecular Probes Inc. (Eugene, OR, USA).^22^NaCl was purchased from New England Nuclear (MA, USA).

### Media used

*Isosmotic medium* (in mM) 137 NaCl, 2.7 KCl, 4.72 Na_2_HPO_4_, 1.50 KH_2_PO_4_, 1.32 CaCl_2_, 1.91 MgSO_4_, 5 glucose, pH 7.4 at 25°C, and 300 mosM.

*Hyperosmotic medium* (in mM) 137 NaCl, 2.7 KCl, 4.72 Na_2_HPO_4_, 1.50 KH_2_PO_4_, 1.32 CaCl_2_, 1.91 MgSO_4_, 5 glucose, 45 mM sucrose, pH 7.4 at 25°C, and 345 mosM.

*Sodium free isosmotic medium* (in mM) 136 Choline chloride, 2.7 KCl, 2.50 K_2_HPO_4_, 1.50 KH_2_PO_4_, 1.01 MgSO_4_, 1.32 CaCl_2_, 5 glucose, pH 7.4 at 25°C, and 300 mosM.

### Treatments

ATP release was induced with 10 μM MST7. Carbenoxolone and probenecid were used as blockers of ATP release and apyrase was used as an extracellular ATP scavenger. Suramin and PPADS were used as generic antagonists of P2 receptors. NF110 was used as a blocker of P2X_1_, P2X_2_ and P2X_3_ receptors. An isosmotic medium, in which NaCl was replaced by choline chloride, was used to study the role of sodium in the kinetics of cell volume and it was denoted as sodium free isosmotic medium.

### Isolation of human erythrocytes

Human blood was obtained by venipuncture from healthy volunteers the day each study was performed. Immediately after blood collection, plasma, platelets and leukocytes were removed by centrifugation (900 x g at 20°C for 3 min). The supernatant and buffy coat were removed and discarded. Isolated red blood cells (rbcs) were resuspended and washed three times in isosmotic medium (see below). Packed rbcs were resuspended in isosmotic medium supplemented with 0.5% bovine serum albumin to the corresponding final hematocrit. All procedures conformed to the Declaration of Helsinki. The Institutional Committee for use and care of laboratory animals (School of Pharmacy and Biochemistry, University of Buenos Aires) approved this study (EXP-UBA: 0032258/14. Approval number: 3398). Written informed consent was given by the donors.

### Extracellular ATP measurements

Extracellular ATP (ATPe) was measured using firefly luciferase, which catalyzes the oxidation of luciferin in the presence of ATP to produce light [37,38].

Two different types of luminometry determinations were performed, *real-time* and *off-line*.

*Real-time* luminometry measurements were carried out with cells laid on coverslips that were mounted in the assay chamber of a custom-built luminometer, as previously described [28]. Because luciferase activity at 37°C is only 10% of that observed at 20°C [39], to maintain full luciferase activity, ATP measurements were performed in a cool chamber acclimated at 20°C. Under the experimental conditions, assay volume did not change during the course of the experiment. Most measurements were performed with 3 10^6^ cells incubated in 40 μL of isosmotic medium. The time course of light emission was transformed into ATPe concentration *versus* time by means of a calibration curve. Increasing concentrations of ATP from 20 to 600 nM were sequentially added to the assay medium from a stock solution of pure ATP dissolved in isosmotic medium.

Results were expressed as [ATPe] at every time point of a kinetic curve (i.e., ATPe kinetics), with [ATPe] expressed as nM for 3 10^6^ cells incubated in 40 μl. Alternatively, increases in [ATPe] were evaluated as the difference between a 20 min post-stimulus [ATPe] and basal [ATPe], and are indicated as ΔATP_20_.

For *off-line* luminometry determinations, ATPe was measured as described by Olearczyk et al. [9]. Rbcs suspensions (20% hematocrit) were exposed to MST7 in iso- and hyperosmotic media, in the absence and presence of probenecid. Afterwards, 4.5 μL of the suspension was used to quantify ATPe concentration. Hemolysis was assessed in paired samples (see method below).

Results were expressed as nM ATPe for 4.4 10^8^ cells incubated in 200 μL.

### Cell volume

#### Epifluorescence microscopy measurements

Under certain conditions fluorescence intensity of fluorophore loaded cells decreases with the increase in fluorophore concentration. This quenching property can be used to measure water volume changes in various cell types by fluorescence microscopy [40], and has been employed by our group to assess volume regulation of human erythrocytes loaded with BCECF [29,32].

Accordingly, the kinetics of cell volume of rbcs before and after addition of MST7 was estimated by the quenching method using BCECF loaded cells [2,29,32]. Rbcs (3 10^6^ cells) were attached to 0.001% poly-D-lysine-coated coverslips and incubated with isosmotic medium containing 5 μM BCECF-AM during 60 min at room temperature. Subsequently, the solution was washed with isosmotic medium to eliminate non-incorporated probe, and the coverslip was mounted on a measuring chamber of an epifluorescence microscope.

Changes in cell water volume were inferred from readings of fluorescence intensity recorded by exciting BCECF at 445 nm, where the fluorochrome is pH-insensitive [2,29,32]. Images were acquired at 30 s intervals during the whole incubation period, except during the first min post stimulus, were images were acquired every second. Values of cell volume were obtained from the fluorescence ratio (*Ft*/*F*0). The value of *F*0 represents the signal obtained from a small circular digital region placed at the image plane of each fluorophore-loaded cell equilibrated with isosmotic medium, whereas *Ft* denotes the fluorescence of the same region of the cell at time *t*. Thus, this measure represents a fractional volume where the initial isosmotic cell volume value is 1 and volume changes are expressed as relative to the initial value. A calibration is needed to convert values of relative fluorescence for each cell to relative volume. Calibration was performed by sequentially exposing cells to assay media of osmolarities (in milliosmolar) 287, 260, 245 or 312, 323 and 340. In Fig A in S1 File Vr calibration curves are shown. Anisotonic media for cell volume calibrations were similar in composition to isosmotic medium, except that NaCl concentration was adjusted appropriately (for hyposmotic media) or appropriate sucrose amounts were added (for hyperosmotic media).

During all of these sequential media exchanges, the *X*, *Y*, and *Z* positions of the microscope field remained unchanged.

Variations of cell volume were expressed as relative volume (Vr) at every time point of a kinetic curve (i.e., Vr kinetics). Alternatively, increases in Vr were evaluated as the Vr value at 5 or 20 min post stimulus, and are indicated as Vr_5_ and Vr_20_ respectively.

#### Hematocrit measurements

The hematocrit is the relative volume (%) occupied by rbcs in blood. Therefore, in a suspension of erythrocytes, hematocrit changes indicate changes in the volume of these cells.

For the determination of cell volume variation, rbcs suspensions were prepared in isosmotic medium at approximately 20% hematocrit. The cells were then exposed for 2 min to isosmotic medium (control) or MST7 10 μM, in the presence or absence of POM1 (an inhibitor of ectonucleotidases). Rbcs suspensions were then centrifuged (5000 x g at 20°C for 3 min) in an 80 μL capillary and the micro hematocrit was measured in the International Equipment Company Device (Boston, Mass) for hematocrit measurement.

### Sodium determination

Intracellular sodium content (Na^+^i) was measured by capillary electrophoresis [41,42]. Briefly, rbcs (3 10^6^ cells) were attached to 0.001% poly-D-lysine-coated coverslips and exposed to isosmotic medium (basal) or MST7 10 μM for 1, 3 or 5 min. To remove extracellular sodium, cells were washed three times with medium containing Hepes 20 mM, imidazole 15 mM and sucrose, 300 mosM, pH 7.4. LiCO_3_ was added to the assay as internal standard (3 μL, 4 mM) and cells were then lysed by exposure to TCA (4% at 4°C for 30 min). The lysate was centrifuged (18000 x g at 20°C for 5 min) and the resulting supernatant was dried in a vacuum concentrator for 2 hours (Savant^™^ SPD131DDA SpeedVac^™^ Concentrator). The samples were then reconstituted in ultrapure water and stored at 4°C.

Capillary electrophoresis was performed with a Beckman P/ACE MDQ system (Beckman Coulter, Brea, CA, U.S.A.), equipped with a diode array detector. Fused-silica capillaries were 60cm in total length (50 cm from the inlet to the detector) and 75 μm id. The running background electrolyte consisted of Imidazol 5 mM and HIBA 6.5 mM, pH 3.0. Runs were carried out in normal mode (cathode at the outlet side, 20 kV) and detection was indirect at 254 nm.

Results were expressed as relative intracellular sodium content, where sodium basal value is 1 and sodium changes are expressed as relative to the initial value.

### Sodium uptake

Suspensions of rbcs (1% hematocrit) were incubated with isosmotic RBC medium containing ^22^Na^+^ (6 μci/mL) in the absence of presence of MST7, and in the absence and presence of NF110. Then, for each experiment, a 50 μL aliquot was poured on top of a 500 μL solution containing 40% phtalic acid bis (2-ethyl-hexyl) ester and 60% di (n-butyl) phtalate, and centrifuged 1 min at 2000x g using an Eppendorf centrifuge. This procedure allowed to separate the extracellular medium, which remained on top of the phtalate solution, from the rbcs, which sedimented forming a pellet.

The pellet was dissolved in 2 mL of 0.5 M NaOH and radioactivity was measured by Cerenkov effect. Results were expressed as nanoequivalents Na^+^/10^6^ cels/min ± SEM.

### Hemolysis measurements

For *real-time* luminometry, hemolysis was evaluated by two methods. First, in the same experiments carried out to evaluate cell volume (see Epifluorescence microscopy measurements above), the kinetics of viability could be assessed in BCECF loaded cells by quantifying the retention of the intracellular fluorophore. A steep, acute loss of fluorophore was interpreted as cell death.

Second, an enzymatic method was used to detect microquantities of free hemoglobin [43].

Experiments with hemolysis > 0.02% were discarded.

In a few experiments designed to assess ATPe by off-line luminometry paired samples were taken to assess hemolysis. Samples were centrifuged at 3000 × g at 20°C for 3 min and the presence of free hemoglobin in the supernatant was determined by light absorption at a wavelength of 405 nm. No experiments were discarded.

### Data analysis

Statistical significance was determined using one-way analysis of variance followed by a Turkey-Kramer test of multiple comparisons. A p value < 0.05 was considered significant. Numbers of determinations (n) from independent preparations (N) are indicated. For experiments on the time course of Vr, 20–30 cells from 3, 4 or 5 independent preparations were used.

Results were also analyzed by means of a mathematical model described in S1 File. To perform the simulations of the model, Eqs. A-M were numerically integrated, employing the Euler method. Unless otherwise specified, the numerical values of the parameters employed for the simulations were those shown in Table A in S1 File. The suitability of different variants of the model was analyzed by means of model fit to experimental data, and the Akaike criterion [44].

## Results

This section is divided into “experimental results”, describing the results of experiments monitoring changes in cell volume (Vr) and ATPe concentration ([ATPe]) under various conditions, and “theoretical results”, describing the simulations and predictions derived from a mathematical model accounting for the dynamic interaction between Vr and [ATPe].

### Experimental results

#### Kinetics of both [ATPe] and Vr of MST7 exposed rbcs

**Fig 1:**
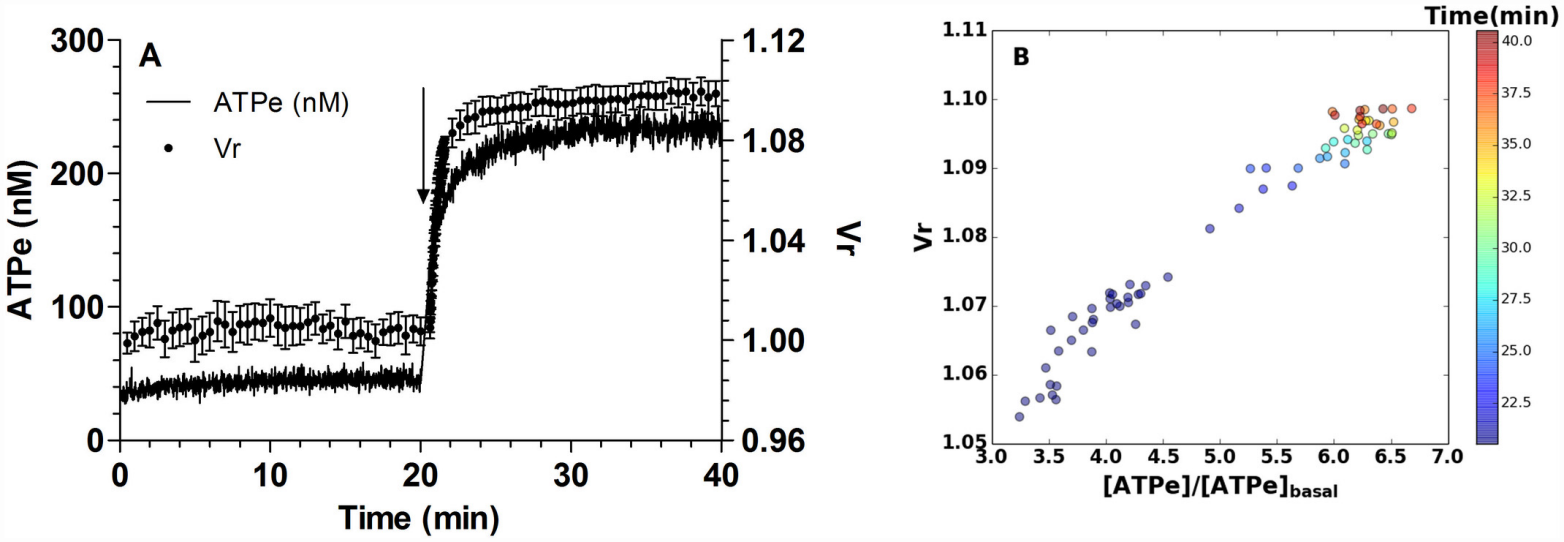
ATPe and Vr kinetics of MST7-exposed rbcs. (A)*Time course of [ATPe] and relative cell volume (Vr) of rbcs exposed to MST7*. Rbcs were incubated 20 min in isosmotic medium (300 mosM), after which 10 μM MST7 was added. ATPe concentration was expressed in nM for 3 10^6^ cells assayed in 40 μL of medium (black line, N = 9, n = 15). Vr is a dimensionless quantity; values are expressed as means ± SE of 20–30 rbcs (●, N = 6). Calibration of Vr was performed at the end of each experiment by sequentially exposing rbcs to assay media with the following osmolarities (in mosM) 298, 287, 260 and 245 (see Fig A in S1 File). Calibration of [ATPe] was carried out using fixed concentrations of exogenous ATP dissolved in isosmotic medium (not shown). The arrow indicates exposure to 10 μM MST7. Numbers of determinations (n) from independent preparations (N) are indicated. (B) *Correlation between Vr and [ATPe]*. Data from A was plotted as Vr against [ATPe] at different times of MST7 exposure. [ATPe] was expressed relative to the basal ATPe concentration measured in the pre-stimulus phase. Colors for symbols reflect the time after MST7 addition according to the time scale shown on the right.

In Fig 1 a quantification of the time dependent changes in [ATPe] and in Vr is shown, i.e., the kinetics of ATPe and Vr.

At any time, ATPe kinetics depends on the rates of ATP release (increasing [ATPe]) by one or more conduits and ATPe hydrolysis (decreasing [ATPe]) by ectoATPase activity.

In the absence of MST7, values of [ATPe] and Vr remained steady at 46 ± 9 nM and 1.003 ± 0.005, respectively.

Addition of 10 μM MST7 promoted an acute increase in [ATPe], amounting 234 ± 35 nM at 20 min post-stimulus, and a fast 10% swelling (Vr_20_ = 1.10 ± 0.01) (Fig 1A).

A correlation plot of Vr vs [ATPe] of MST7 exposed rbcs showed a positive, almost linear correlation between these two variables, with Pearson coefficient amounting to 0.983 (Fig 1B).

Thus, a series of experiments were run to test for mutual relationships between [ATPe] and Vr.

#### Effect of [ATPe] on Vr kinetics

The effect of [ATPe] on Vr kinetics was evaluated by either inhibiting ATP efflux with CBX and PBC (two well-known pannexin 1 inhibitors) or removing ATPe with apyrase (Apy), an ATPe scavenger.

**Fig 2:**
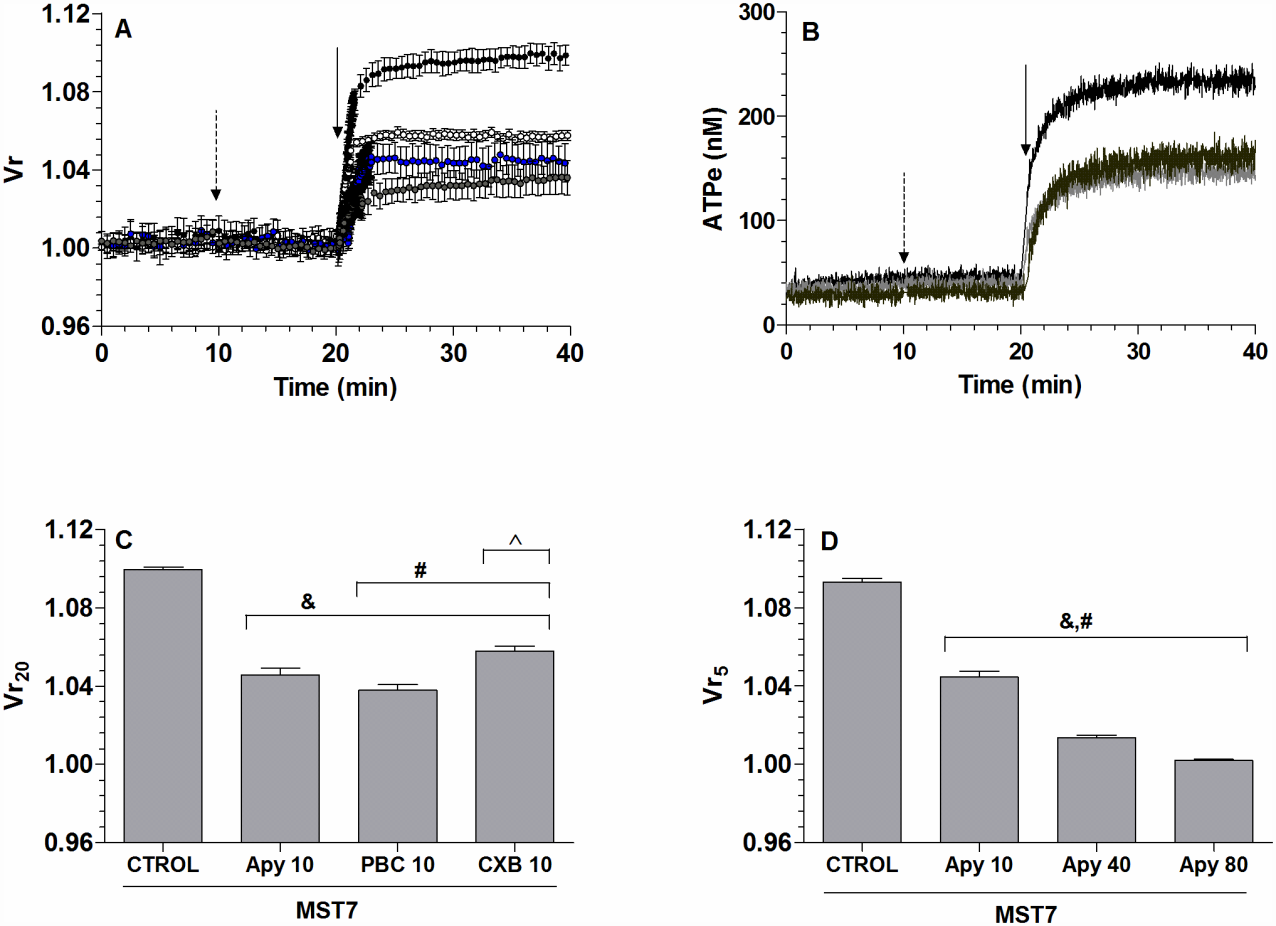
Effect of ATP release on the kinetics of Vr and [ATPe]. (A) *Time course of Vr for rbcs exposed to MST7*. BCECF-loaded rbcs were incubated in 200 μL isosmotic medium in the absence or presence of 10 μM CBX (○), 10 μM PBC (●), or 10 U/mL apyrase (Apy, ●). After 20 min, 10 μM MST7 was added. Results are means ± SE of 20–30 rbcs for CBX (N = 4), PBC (N = 3) and apyrase (N = 3). The dashed arrow indicates addition of treatment and the full arrow indicates exposure to 10 μM MST7. Numbers of independent preparations (N) are indicated. (B) *Time course of [ATPe] for rbcs exposed to MST7*. Rbcs were incubated in 40 μL isosmotic medium in the absence or presence of 10 μM CBX (light grey line, N = 5, n = 8) or 10 μM PBC (dark grey line, N = 3, n = 5). After 20 min, 10 μM MST7 was added. The dashed arrow indicates addition of blockers and the full arrow indicates exposure to MST7. Numbers of determinations (n) from independent preparations (N) are indicated. (C) *Degree of swelling derived from results shown in A*. Results are given as Vr_20_, i.e., the Vr value at 20 min post MST7 exposure. Results are means ± SEM (&, p < 0.001 *versus* CTROL, MST7 alone; #, p < 0.01 *versus* Apy; ^, p < 0.001 *versus* PBC10). (D) *Effect of apyrase on swelling*. BCECF-loaded rbcs were pre-incubated for 10 min with 10, 40 or 80 U/mL Apy, followed by exposure to 10 μM MST7 for 5 min. The degree of swelling was expressed as Vr_5_, i.e., the Vr value at 5 min post MST7 exposure. Results are means ± SEM (&, p < 0.001 *versus* CTROL, MST7 alone; #, p < 0.001 among apyrase concentrations). CBX = carbenoxolone; PBC = probenecid, Apy = apyrase. Calibrations of Vr and [ATPe] were performed as in Fig 1.

CBX and PBC, which partially inhibited the kinetics of ATPe (Fig 2B), induced a reduction of swelling (Fig 2A), with Vr_20_ being 40–60% of the control value in the absence of inhibitors (Fig 2C).

Under 10 U/mL apyrase exposure, MST7 dependent swelling was partially reduced (Fig 2A), with Vr_20_ being 60% lower than that observed in the absence of apyrase. Additional short term experiments were run to test the effect of apyrase concentration on MST7-dependent swelling. Results were recorded at 5 min post-stimulus, i.e., Vr_5_ (Fig 2D). It can be seen that higher apyrase concentrations led to a higher degree of swelling inhibition, with swelling being completely blocked at 80 U/mL apyrase.

#### Effect of Vr on [ATPe] kinetics

Since swelling is a well-known inducer of ATP release, we wondered whether part of the effect caused by MST7 on ATPe kinetics could be due to the acute Vr increase observed in Fig 1A.

**Fig 3:**
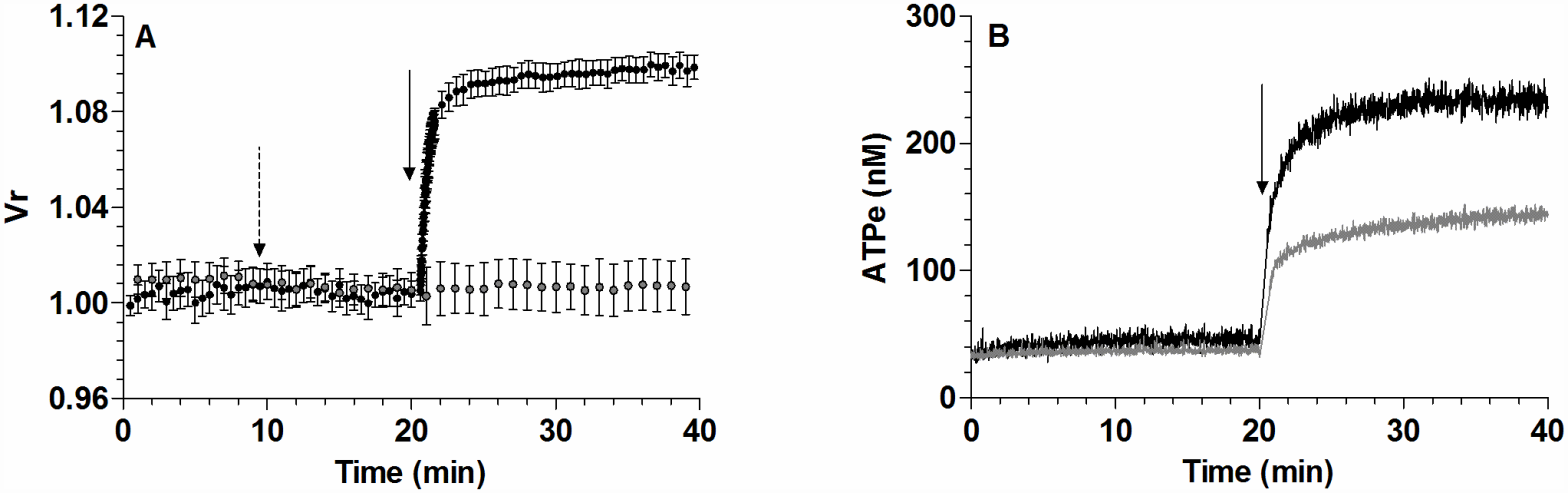
Effect of hyperosmotic medium on kinetics of Vr and [ATPe]. (A) *Time course of Vr for rbcs exposed to MST7*. BCECF-loaded rbcs were incubated 20 min in 200 μL of isosmotic medium (300 mosM), followed by exposure to 10 μM MST7 dissolved in isosmotic medium (●) or in hyperosmotic medium (●, 345 mosM). Results are means ± SE of 20–30 rbcs (N = 6) for isosmotic medium and 20–30 rbcs (N = 4) for hyperosmotic medium. Calibration was performed at the end of each experiment by sequentially exposing rbcs to assay media with the following osmolarities (in mosM) 287, 260, 245 or 312, 323 and 340 (see Fig A in S1 File). The arrow indicates exposure to 10 μM MST7. Numbers of independent preparations (N) are indicated. (B) *Time course of [ATPe] for rbcs exposed to MST7*. Rbcs were stimulated with 10 μM MST7 dissolved in isosmotic medium (black line; N = 9, n = 15) or in hyperosmotic medium (gray line; N = 3, n = 5). [ATPe] was expressed in nM for 3 10^6^ cells assayed in 40 μL of medium. The arrow indicates exposure to stimulus. Numbers of determinations (n) from independent preparations (N) are indicated. Calibrations of Vr and [ATPe] were performed as in Fig 1.

Thus, MST7 was prepared in a hyperosmotic medium (345 mosM), which fully blocked swelling of rbcs (Fig 3A). Under this condition ATPe kinetics was 40% inhibited (ΔATP_20_ = 108 ± 14) with respect to that observed with MST7 dissolved in isosmotic medium (Fig 3B).

#### Effect of P receptor activation on Vr and ATPe kinetics

Results above showed that, under MST7 exposure, increases of [ATPe] induced an increase in Vr which, in turn, triggered further increases of [ATPe]. We tested whether this process occurred via activation of P receptors.

**Fig 4:**
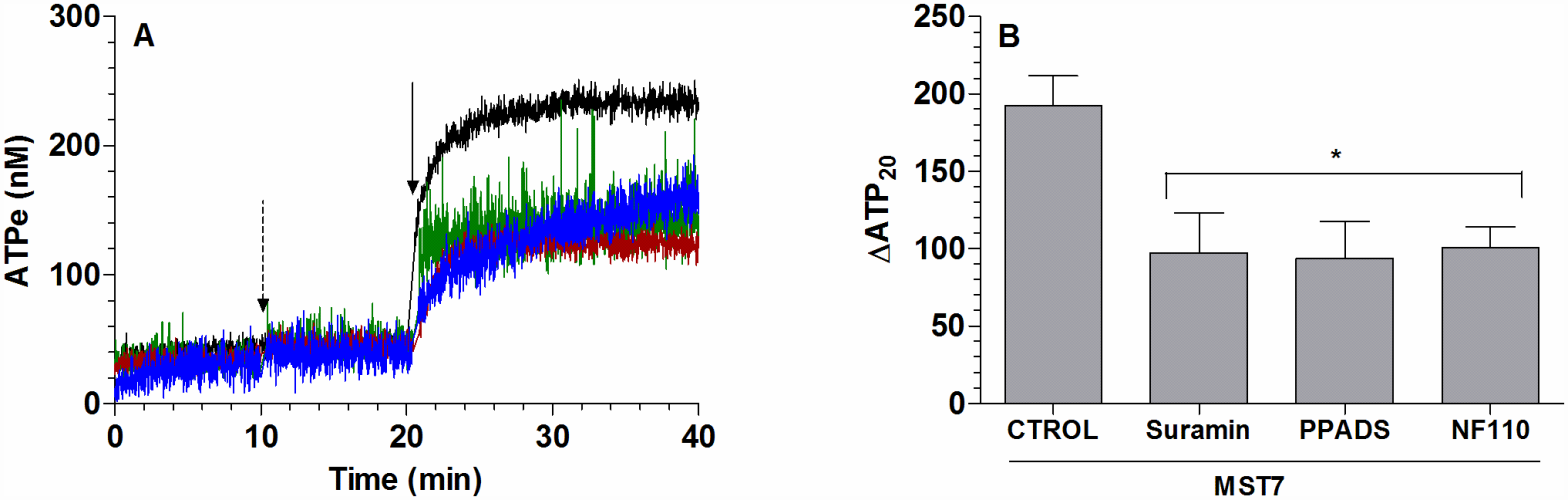
Effect of P receptors on ATPe kinetics. (A) *Time course of [ATPe] for rbcs exposed to MST7*. Rbcs were incubated in 40 μL medium in the absence or presence of the following P receptor antagonists: 100 μM suramin (green line, N = 5, n = 6), 100 μM PPADS (brown line, N = 5, n = 6) or 10 μM NF110 (blue line, N = 3, n = 5). After 20 min, 10 μM MST7 was added. The dashed arrow indicates addition of blockers and the full arrow indicates exposure to MST7. Numbers of determinations (n) from independent preparations (N) are indicated. ATPe was expressed in nM for 3 10^6^ cells assayed in 40 μL of medium. (B) *Values of [ATPe] increase using data from A*. Values are expressed as ΔATP_20_, i.e., the difference between [ATPe] at 20 min post stimulus and basal [ATPe]. Results are means ± SEM (*, p < 0.05 *versus* CTROL, MST7 alone) and are expressed in nM for 3 10^6^ cells assayed in 40 μL of medium. Calibration of [ATPe] was performed as in Fig 1.

First, MST7 dependent ATPe kinetics was tested in the presence of 100μM suramin and 100 μM PPADS, two generic inhibitors of P receptors, and 10 μM NF110, an inhibitor of subtypes 1, 2 and 3 of P2X receptors (Fig 4A). Similar kinetics were found among the three treatments, although under suramin and PPADS exposure, ATPe kinetics saturated at 154–160 nM, while in the presence of NF110 a slight, almost linear component of ATPe increase could be seen at t > 21 min. Despite these differences, in all cases a partial inhibition of ATPe kinetics was observed, yielding similar values of ΔATP_20_ at approximately 50% of the control condition (Fig 4B).

Since NF110, as a P2X_1-3_ blocker, is more specific than suramin and PPADS, we evaluated the effect of this compound on Vr kinetics. Mechanistically, P2X activation may induce sodium influx, yielding an increase of intracellular osmolarity, followed by water influx and the corresponding increase in Vr.

Thus, MST7-induced Vr kinetics was monitored under two treatments potentially altering sodium homeostasis, i.e., pre-incubation with NF110, and exposure to sodium free isosmotic medium (i.e., isosmotic medium where sodium was replaced by choline chloride).

**Fig 5:**
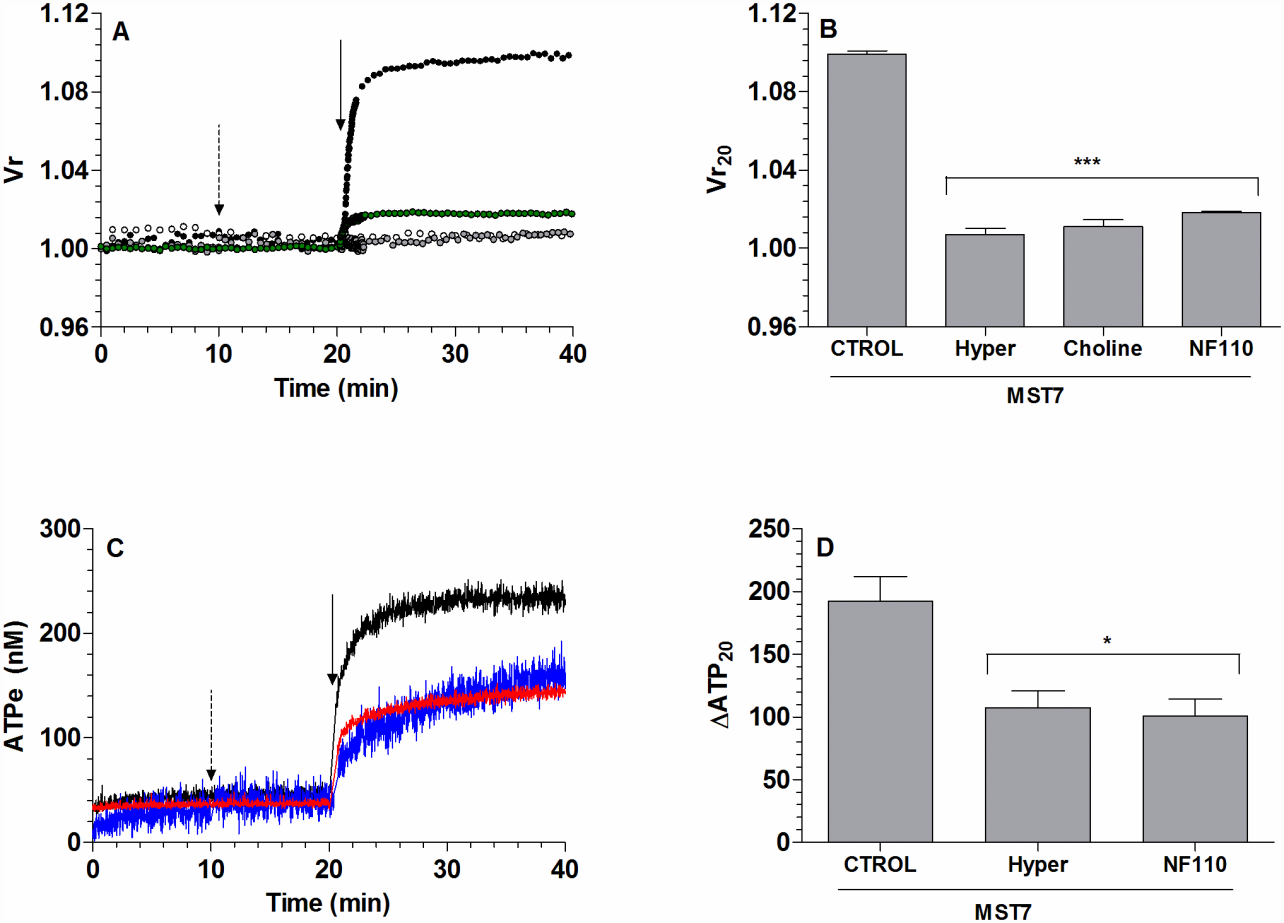
Effect of P2X receptor and sodium on the kinetics of Vr and ATPe. (A) *Vr kinetics*. BCECF-loaded rbcs were incubated in isosmotic medium. After 10 min the medium was replaced by a sodium free isosmotic medium (●) or an isosmotic medium containing 10 μM NF110 (●). Kinetics of Vr for rbcs exposed to MST7 in isosmotic (●) and hyperosmotic (○) media are shown for comparison. Calibration was performed as in Fig 1. Results are means ± SE of 20–30 rbcs (isosmotic medium, N = 6; NF110, N = 4; Choline, N = 3) and 30–40 rbcs for hyperosmotic medium (N = 4). The dashed arrow indicates addition of blocker and the full arrow indicates addition of MST7. (B) *Degree of swelling obtained from A*. Values are expressed as Vr_20_, i.e., the Vr value at 20 min post stimulus. Results are means ± SEM (***, p < 0.001 *versus* CTROL, MST7 alone). (C) *ATPe kinetics*. Rbcs were pre-incubated with 10 μM NF110 (blue line, N = 3, n = 5) for 10 min before exposure to 10 μM MST7. ATPe kinetics for MST7 in isosmotic (black line) and hyperosmotic (red line) media are shown for comparison. Results are expressed in nM for 3 10^6^ cells in 40 μL assay medium. The dashed arrow indicates addition of blocker and the full arrow indicates addition of MST7. (D) *Values of [ATPe] increase using data from C*. Values are expressed as ΔATP_20_, i.e., the difference between [ATPe] at 20 min post stimulus and basal [ATPe]. Results are means ± SEM. (*, p < 0.05 *versus* CTROL, MST7 alone) and are expressed in nM for 3 10^6^ cells assayed in 40 μL of medium. Calibrations of Vr and [ATPe] were performed as in Fig 1.

These treatments caused a strong reduction of Vr kinetics (Fig 5A), with Vr_20_ being about 20% (NF110) and 10% (sodium free isosmotic medium) of the control condition (Fig 5B).

On the other hand, hyperosmotic medium fully blocked swelling, and reduced ATPe kinetics by 40% (Fig 5).

**Fig 6:**
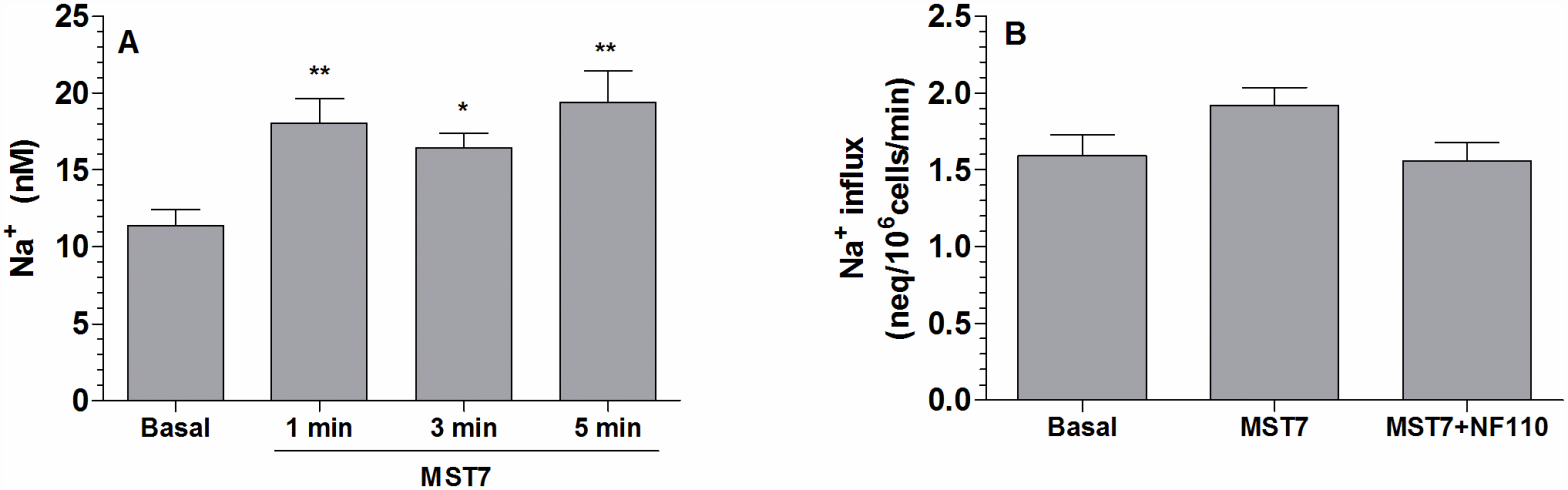
Effect of MST7 on intracellular sodium content. (A) Rbcs were exposed to isosmotic medium in the absence or presence of 10 μM MST7 for 1, 3 or 5 min. Cells were then lysed and sodium content was measured by capillary electrophoresis. Results (Na^+^) were expressed in mM. Values are means ± SEM (*, p < 0.05 *versus* Basal) (N = 3, n = 6–12). (B) Sodium uptake in the absence of MST7 (basal) and in the presence of MST7, both with and without pre-exposure to NF110. Experiments were run for 1 min following exposure to RBC medium containing ^22^NaCl ± 10 μM MST7. 10 μM NF110 was given 10 min before addition of radioactive label. Numbers of determinations (n) from independent preparations (N) are indicated.

As a final test for the involvement of intracellular Na^+^ in the responses of Vr and ATPe, Na^+^ content and Na^+^ influx were measured in rbcs. As seen in Fig 6, exposure of cells to MST7 led to an approx. 60% increase in Na^+^ content, which remained relatively elevated for at least 5 min post-stimulus (Fig 6A). Exposure of rbcs to MST7 for 1 min increased Na^+^ by 18% with respect to basal values. This increase was fully blocked by pre-exposure to NF110 (Fig 6B).

#### Validation of the swelling response

**Fig 7:**
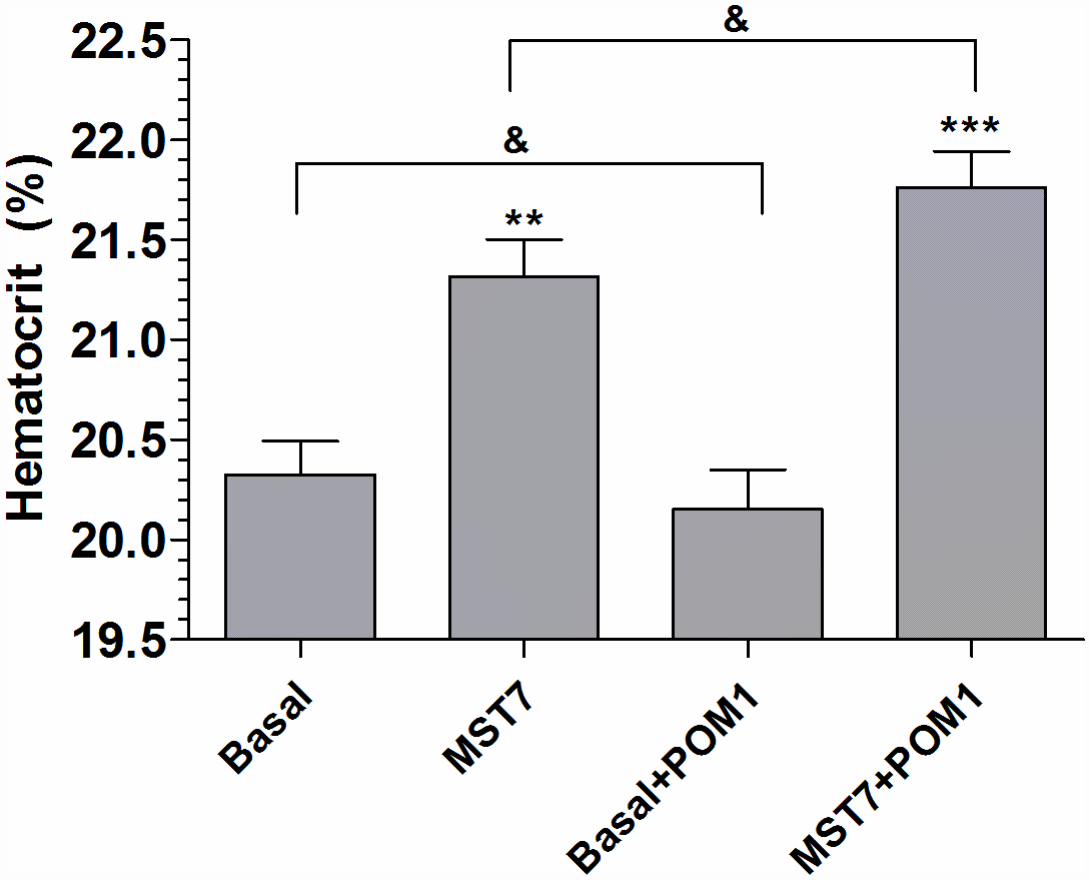
Effect of MST7 on hematocrit of rbcs. Rbcs were held in suspension in isosmotic medium at 20% hematocrit, and exposed 10 min to isosmotic medium in the absence and presence of POM1 (an ectoATPase inhibitor) followed by exposure to 10 μM MST7 for 2 min. Hematocrits were then determined, and expressed as percentage (%). Results are means ± SEM (N = 5, n = 46) (***, p < 0,001 versus Basal; **, p < 0,05 *versus* Basal + POM1; &: not significant). Numbers of determinations (n) from independent preparations (N) are indicated.

We checked whether the swelling response of Fig 1 A was compatible with an increase in Vr in high density rbcs suspensions, estimated by the hematocrit. Rbcs were incubated 10 min in isosmotic medium in the absence and presence of POM1 (an ectonucleotidase inhibitor) followed by exposure to 10 μM MST7 for 2 min. POM1 did not affect hematocrit in the absence of MST7. Hematocrit values increased 5 ± 0.2% with MST7 and 8 ± 0.2% with MST7 + POM1 (Fig 7).

#### Validation of the ATPe response

**Fig 8:**
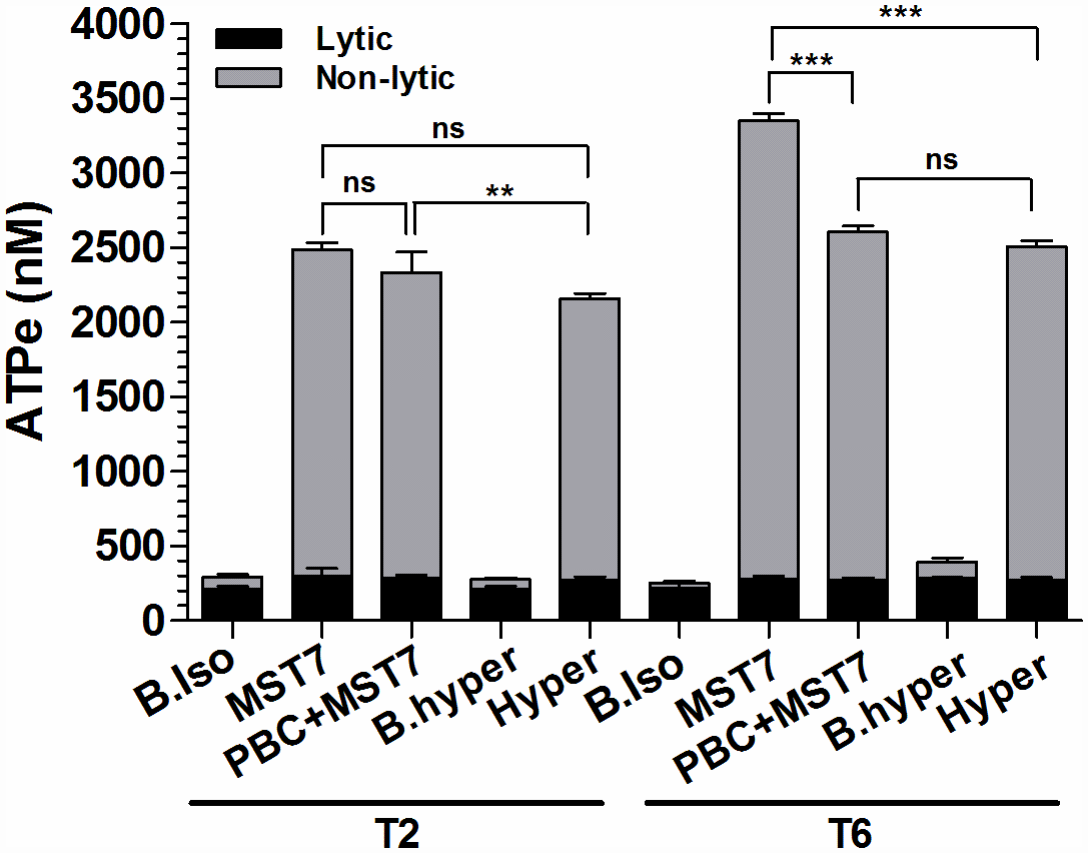
ATPe concentration of a suspension of rbcs exposed to MST7. Rbcs were held in suspension in isosmotic medium (300 mosM) at 20% hematocrit and exposed to 10 μM MST7 for 2 and 6 min (T2 and T6 respectively). MST7 was added in the absence of additional treatments (MST7), or in the presence of hyperosmotic medium (Hyper, 345 mosM) or 10 μM probenecid (PBC). Controls were run in the absence of MST7, both in isosmotic (B.iso) as well as hyperosmotic (B. hyper) media. Assessment of hemolysis for each sample allowed to calculate the lytic contribution to ATPe concentration. Results are means of N = 3, n = 5. Numbers of determinations (n) from independent preparations (N) are indicated.

In experiments of Fig 8. we used 20% hematocrit suspensions to assess ATPe and hemolysis simultaneously. Rbcs were held in suspension in isosmotic medium (300 mosM) at 20°C and exposed to 10 μM MST7 for 2 and 6 min. MST7 was added in the absence of additional treatments (MST7), or in the presence of hyperosmotic medium (Hyper, 345 mosM) or 10 μM probenecid (PBC).

In qualitative agreement with the online measurements, results showed an acute release of ATP induced by MST7, which was partially reduced by PBC. Hyperosmotic medium per se did not produce ATP release, but it partially inhibited MST7-induced ATP release. Thus, there was a component of ATP release which did not depend on swelling. Parallel assessment of hemolysis (Table 1) allowed to calculate the lytic contribution to ATPe concentration. A basal hemolysis of about 0.05% was observed, which did not increase with time, neither in controls, nor in any other treatment.

**Table 1:** Hemolysis assessed for off-line ATPe determinations.

	T2	T6
	Lysis (%)	Lysis (%)
B. Iso	0.04 ± 0.004	0.04 ± 0.008
MST7	0.05 ± 0.010	0.05 ± 0.004
PBC + MST7	0.05 ± 0.003	0.05 ± 0.003
B. hyper	0.04 ± 0.006	0.05 ± 0.001
Hyper + MST7	0.05 ± 0.003	0.05 ± 0.004

In experiments designed to assess ATPe by off-line luminometry, paired samples were taken to assess hemolysis. Rbcs suspensions (20% hematocrit) were exposed to MST7 in iso- (iso) and hyperosmotic (hyper) media, in the absence and presence of probenecid (PBC). Hemolysis was measured 2 min (T2) and 6 min (T6) post-treatment. Basal measurements (B) refer to a control condition in the absence of MST7. Results are expressed as percentage (%) of hemolysed rbcs with respect to the total number of rbcs, and are mean values ± SEM of 5 determinations from 3 independent preparations.

#### Theoretical results: modeling of ATPe and cell volume kinetics of MST7-stimulated rbcs

A mathematical model was built to account for the dynamic interaction of Vr and [ATPe]. A detailed mathematical description of the model is given in S1 File, with values of best fit for the parameters given in Table A in S1 File.

#### Modeling Vr kinetics and its dependence with [ATPe]

**Fig 9:**
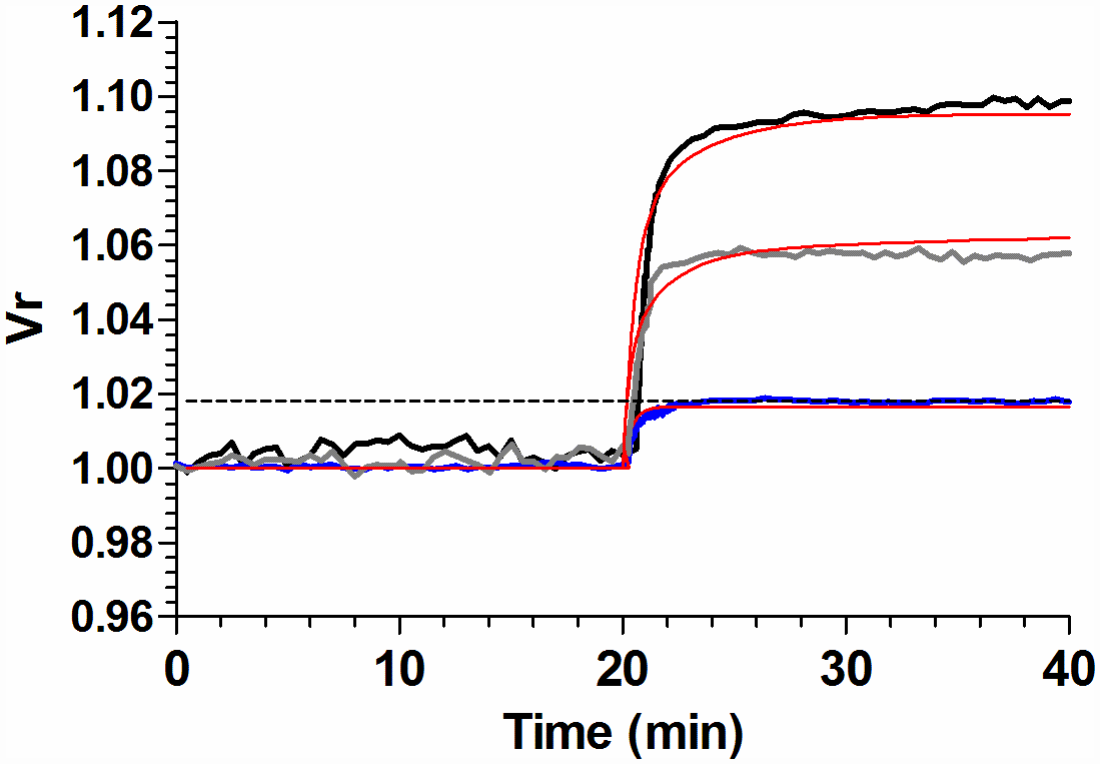
Model simulations of Vr kinetics in the absence and presence of blockers. Model dependent fit to experimental Vr kinetics in the absence of blockers (control condition), and in the presence of 10 μM CBX or 10 μM NF110. Fitting was performed simultaneously to the three experimental conditions. MST7 was added at t = 20 min. Black, grey and blue lines correspond to experimental data of Figs 1A, 2A and 5A, respectively. Red lines show the best fitting curves to experimental data. The dashed line indicates the critical value of Vr^c^ above which ATP release is triggered by swelling. Model for Vr kinetics is encoded in Eqs. A-B of S1 File, with values of best fit for the parameters given in Table A in S1 File.

Experimental results showed that, for rbcs incubated with MST7 alone (control), MST7 + CBX, and MST7 + NF110, acute non linear increases of Vr were observed (Fig 9). Vr kinetics was described by a double exponential function of time, with a fast phase spanning about 80% of total Vr change.

For cells under MST7 ± CBX, the amplitude of the fast phase depended almost linearly on [ATPe], whereas for MST7 + NF110, Vr changes did not depend on P2X activation by [ATPe]. Results of Fig 9 showed a good fit of the model to experimental data under the three different conditions, using a single set of best fit values for the parameters (see Table A in S1 File).

#### Modeling ATPe kinetics and its dependence with Vr

Exposure to CBX induced a partial inhibition of ATPe kinetics (Fig 2B). This was interpreted in the model by assuming that ATP permeability (P_ATP_) is mediated by two conduits, i.e., two ATP permeabilities denoted as P_ATP_^1^ and P_ATP_^2^. To account for swelling activating ATP efflux, the best fit exponential function describing Vr kinetics was included in the ATPe kinetics model via a threshold function. That is, swelling activated P_ATP_ only when Vr was equal or higher than a critical value Vr^c^.

**Fig 10:**
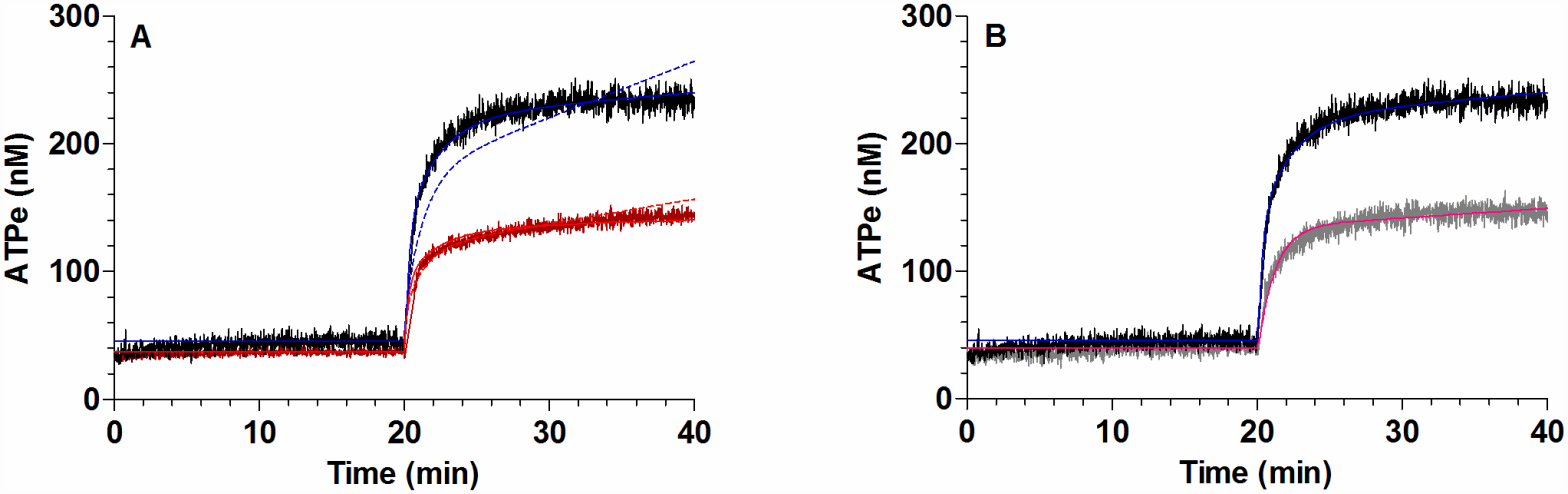
Model simulations of [ATPe] kinetics. (A) The lines represent the best fit of the model to experimental ATPe kinetics for rbcs exposed to MST7 in isosmotic and hyperosmotic media. Two ATP conduits were assumed to mediate ATP efflux. The model was run assuming that one (dashed lines) or both conduits (continuous lines) were sensitive to swelling. Experimental data was obtained from Fig 3B, and showed only for comparison. (B) The lines represent the best fit of the model to experimental ATPe kinetics for rbcs exposed to MST7 in the absence and presence of 10 μM CBX. Two Vr-sensitive ATP conduits were assumed. The model was run assuming that CBX blocked one ATP conduit and partially inhibited the second conduit. Experimental data was obtained from Fig 2B, and showed only for comparison. MST7 is added at t = 20 min. The model is described in S1 File, with best fitting values shown in Table A in S1 File.

We first tested whether swelling affected one or both ATP permeabilities. First, we fitted the model to the experimental ATPe kinetics in the presence of swelling (*i*.*e*., MST7 in isosmotic medium) or in the absence of volume change (*i*.*e*., MST7 in hyperosmotic medium). Simulations showed that a model where only one ATP conduit is Vr sensitive did not fit well to experimental data. According to the Akaike criterion [44], a much better fit was obtained when both conduits were affected by Vr changes (Fig 10A).

Thus, assuming two Vr sensitive ATP conduits, we then investigated ATPe kinetics in the absence and presence of CBX. We first considered that CBX completely prevented the activation of one conduit, while leaving the other conduit unaffected. In this case fitting to experimental data was poor (not shown); a much better fit was attained by assuming that CBX not only blocked one conduit (*P*_*ATP*_^*2*^*)* but also affected the other one (Fig 10B).

Once a good fit of the model was attained in the absence and presence of Vr change (Fig 10A), and in the absence and presence of CBX (Fig 10B), the corresponding ATP permeabilities for each conduit could be predicted.

**Fig 11:**
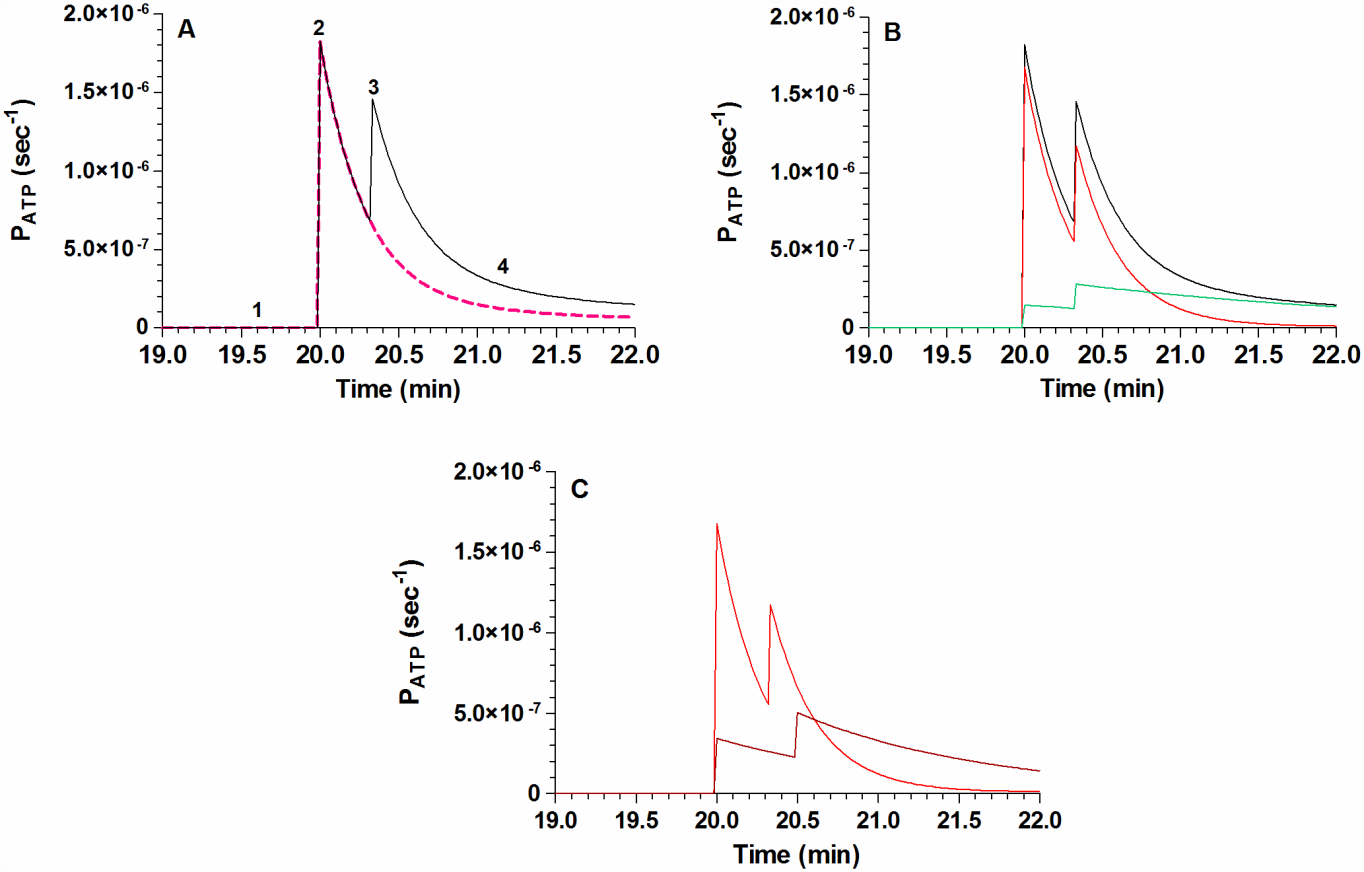
Model prediction of ATP permeability (P_ATP_). Following model dependent fit to the experimentally observed ATP kinetics (Fig 10), the model predicted the corresponding permeabilities of ATP that mediate ATP exit. (A) Kinetics of P_ATP_ for rbcs exposed to MST7 in isosmotic medium (continuous line) or in hyperosmotic medium (dashed line). Labels 1–4 were added to divide the continuous time profile of P_ATP_ into four discrete phases: 1 = basal level of P_ATP_. 2 = P_ATP_ activation by exposure to MST7. 3 = P_ATP_ activation by swelling, which occurred only in isosmotic medium (continuous line). 4 = time dependent inactivation of P_ATP_. (B) Kinetics of P_ATP_ for each ATP conduit (P_ATP_^1^ and P_ATP_^2^, red and green, respectively) and their sum (black). (C) Kinetics of P_ATP_^1^ in the absence of CBX (red, as in B), or in its presence (brown). According to the model, P_ATP_^2^ is zero in the presence of CBX. Model parameters are shown in Table A in S1 File.

In Fig 11A, labels 1–4 were added to analyze the *P*_*ATP*_ time profile in four discrete phases. Prior to stimulus, *P*_*ATP*_ was extremely low (phase 1); it amounted to (0.9 ± 0.2)10^−9^ sec^-1^. Addition of MST7 led to a 1960-fold increase in *P*_*ATP*_, followed by a short lasting decay (phase 2). Then, a second activation of *P*_*ATP*_ was triggered (phase 3) when Vr surpassed the value of Vr^*c*^ (see Table A in S1 File). Finally P_ATP_ inactivated with time (phase 4).

When the model did not include the effects of swelling on P_ATP_, the second activation peak was lost (Fig 11A). The predicted ATP permeabilities of each conduit are shown in Fig 11B.

When CBX was present, *P*_*ATP*_^*2*^ was considered blocked. Under this condition *P*_*ATP*_^*1*^ was partially reduced, a result consistent with an approx. 4-fold reduction in k1 of Eq. I (see Table A in S1 File), and the second activation peak was right shifted (Fig 11C).

**Fig 12:**
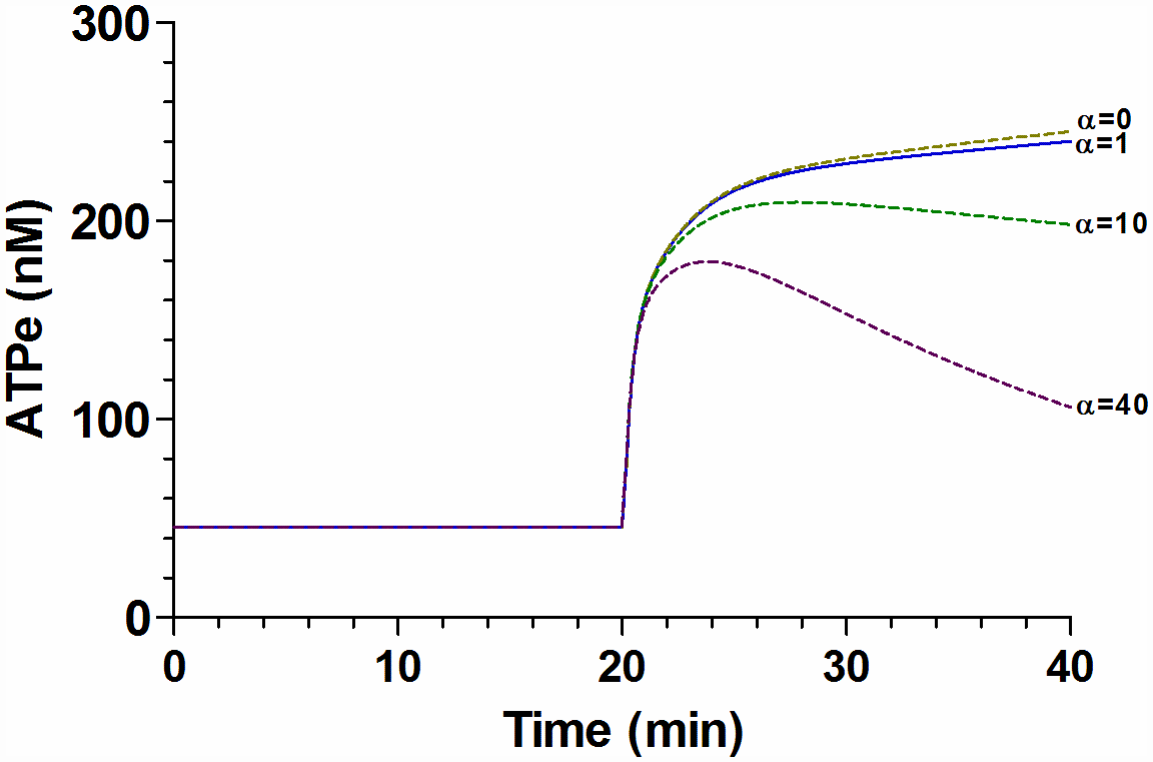
Model prediction for the effect of ectoATPase activity on ATPe kinetics. The effect of ectoATPase activity on ATPe kinetics was evaluated by multiplying k_ATP_, i.e., the parameter estimating ectoATPase activity (see S1 File), by a factor α. The continuous line shows the simulation of the best fit model for ATPe kinetics under MST7 exposure, i.e., α = 1, with k_ATP_ = 1.98 10^−5^ s^-1^. Predictions (dashed lines) were made for α = 0 (i.e., no ectoATPase activity), 10 or 40. MST7 is added at t = 20 min. Model parameters and their best fitting values are shown in Table A in S1 File.

In Fig 12 we show the results of using the best fit model to predict the effect of ectoATPase activity, estimated by k_ATP_ (Eq. D of S1 File), on the simulated ATPe kinetics. Predictions were made for the reference value of k_ATP_ multiplied by a factor α. The control condition is, per definition, α = 1, were k_ATP_ = 1.98 10^−5^ s^-1^ (Table A in S1 File), and the predicted ATPe kinetics coincides with that of Fig 10A and 10B. When α = 0, k_ATP_ is blocked (i.e, no ectoATPase activity). It can be seen that the corresponding effect on ATPe kinetics was very small. For α = 10 and α = 40, the predicted ATPe kinetics departed from the control curve (i.e., that with α = 1).

## Discussion

A major finding of this study was the elucidation of the dynamic interaction between [ATPe] and Vr of rbcs. The peptide MST7 was used as a robust inducer of ATP release [5,10,11,45,46], which simultaneously caused isosmotic cell swelling in the absence of hemolysis. As we discussed below, P2X receptors proved to be key components of a system transducing the observed changes in [ATPe] into changes in Vr.

### ATPe kinetics and its dependence on swelling

Exposure of rbcs to an isosmotic medium containing MST7 induced a small and acute Vr increase (Fig 1A). The simultaneous activation of ATP efflux was partly due to this swelling. This was corroborated by incubating cells in MST7 dissolved in a hyperosmotic medium, so as to fully prevent Vr change. Under this condition ATPe kinetics was strongly inhibited (Fig 3). Thus, ATP release of rbcs was highly sensitive to swelling, since under MST7 exposure, a ≈ 10% Vr increase accounted for ≈ 40% of the accumulated [ATPe].

A mathematical model was built to analyze ATPe kinetics and the effects of swelling.

Although in principle ATPe kinetics depends on ATP efflux and ATPe hydrolysis, the latter is almost negligible (see “*Effect of ectoATPase activity on ATPe kinetics”* below). On the contrary, ATPe efflux is significant, and is mediated by a permeability P_ATP_. Since CBX fully blocks pannexin 1 of rbcs [2,29] but only partially inhibited MST7-dependent ATP efflux (Fig 2), the model assumes that ATP release requires two conduits, i.e., two permeabilities, denoted as *P*_*ATP*_^*1*^ and *P*_*ATP*_^*2*^. By fitting the model to the experimental ATPe kinetics in the presence of swelling (*i*.*e*., MST7 in isosmotic medium) or in the absence of volume change (*i*.*e*., MST7 in hyperosmotic medium), we observed that the best fit required both *P*_*ATP*_^*1*^ and *P*_*ATP*_^*2*^ to be sensitive to swelling (Fig 10).

Once a good fit of the model was attained in the absence and presence of Vr change, and in the absence and presence of CBX (Fig 10), the corresponding global permeability of ATP and the permeabilities for each conduit could be predicted.

P_ATP_ was extremely low in the absence of stimulus, a condition compatible with one or more ATP channels dwelling in low conductance states [47]. Exposure to MST7 triggered an approx. 2000 fold increase of P_ATP_, followed by a short lasting decay. This is consistent with MST7 acutely activating heterotrimeric Gi protein of rbcs, with subsequent increase in cAMP formation, followed by a series of signaling steps upstream of ATP release [46,48,49].

According to the model, this first transition was not affected by swelling, because Vr laid below a critical value called Vr^*c*^. Then, a second steep increase in *P*_*ATP*_ was triggered when V_r_, which continuously increased during at least 20 sec of the swelling response (Fig 1A), surpassed Vr^*c*^. At later times of MST7 exposure, even when Vr values remained higher than Vr^*c*^ for the rest of the experiment, P_ATP_ progressively inactivated with time.

The kinetic profile of P_ATP_ gave rise to the simulated control curve of ATPe kinetics (i.e., MST7 in isosmotic medium, Fig 10B). Fig 11A also shows a hypothetical situation where P_ATP_ was not affected by swelling, a condition representing exposure of rbcs to MST7 dissolved in hyperosmotic medium. In this case, the predicted strong inhibition of ATPe kinetics (Fig 10A) was due to blockage of the Vr-sensitive second activation peak of P_ATP_ (Fig 11A).

The two conduits showed different kinetic profiles (Fig 11B). During the first minute MST7-dependent ATP release was mainly controlled by P_ATP_^*1*^, whereas P_ATP_^*2*^, with a much lower inactivation kinetics, became important beyond that time point.

A qualitatively similar kinetic pattern of P_ATP_ could also be found forP_ATP_^*1*^ and P_ATP_^*2*^, in that two activation steps coexist with a continuous time dependent inactivation process. In this respect, the activities of pannexin 1 and voltage-gated channels, some of which were postulated to transport ATP, showed transient acute activation, followed by spontaneous attenuation [47,50]. Regarding the second activation peak, pannexin 1 and several anion channels [51–54]were shown to be sensitive to swelling and mechanical stress [34,55,56].

Finally when CBX is present P_ATP_^*2*^wasset to zero. In this case, the model predicted that P_ATP_^1^ (P_ATP-CBX_ of Fig 11C) was partially reduced. In support of these results is the fact that CBX not only fully blocked pannexin 1 (P_ATP_^2^ of the model) in rbcs of humans and other vertebrates [34,57–59], but also inhibited other transporters thought to serve as ATP conduits. Among them are voltage dependent anion channels (VDAC1 in rbcs [50], CALHM1 in taste bud cells [22], or the maxi anion channels [33]. An effect of CBX on ATP release by connexins was discarded, since connexin currents are blocked at much higher concentrations of CBX than that used in this study [60]. Moreover, the presence of connexins was not reported in rbcs [34].

Can MST7 create a pore to act as an ATP conduit?

Under certain conditions, various mastoparan peptides have been shown to form exogenous pores on cell membranes [61]. However, pore formation in our experimental setting is unlikely for several reasons:

1. In experiments of Figs 1A, 2A, 3A and 5A, BCECF loaded rbcs were used. At neutral pH, BCECF is an anion with comparable size as ATP. If MST7 created an exogenous pore, then we would observe an acute drift in fluorescence intensity soon after MST7 exposure. But this drift did not occur.
2. The experimental ATPe kinetics (Figs 1A, 2B, 3B, 4A and 5C) was measured under conditions where:
  - EctoATPase activity was negligible, so that the experimental time dependent profile of ATPe accumulation reflects the permeability of ATP (see “Effect of ectoATPase activity on ATPe kinetics” below).
  - The driving force (ATP transmembrane chemical gradient) of ATP efflux did not change significantly during MST7 exposure.
  Under these conditions, the calculated permeabilities of ATP (Fig 11) were activated and inactivated by time very rapidly. These permeabilities, after a delay, were also briefly activated by swelling. This transient kinetics of activation-inactivation is more compatible with pannexin 1 and anion channels, which exhibit gating properties and, as mentioned above, can be activated by swelling and/or stretching forces [36,60,62].
- Mastoparan at high concentrations (30 min exposure to 50–100 μM) increased the permeability of rbcs membranes, changing the discoid shape to a crenated form [61]. However, using 10 μM MST7 in this study we neither observed crenation, nor shrinking.

### ATPe kinetics and its dependence on P receptor activation

An important aspect of this study concerns the role of P receptors as mediators connecting the parallel changes in Vr and [ATPe].

ATPe kinetics was measured in the absence of inhibitors, and in the presence of suramin or PPADS, two generic inhibitors of P2 receptors, and NF110, an inhibitor of subtypes 1, 2 and 3 of P2X receptors.

In all cases a partial inhibition of ATPe kinetics was observed. The fact that similar values of ΔATP_20_ were obtained in the presence of suramin, PPADS and NF110 (Fig 4) suggests that P2X_1-3_ might be involved in the response. This agrees well with rbcs lacking P2Y receptors with high affinity for ATPe [63]. These cells exhibit a high density of functional P2Y_13_ receptors, which however display a higher affinity for ADP than ATP [63]. In our hands, although P2Y_13_ activation by 2-MeSADP (an ADP analog) significantly decreased ATPe efflux (Fig B in S1 File) this effect is negligible under our experimental setting. This is because even in the presence of50-230nM ATPe (Fig 1A), ADP in assay medium is assumed to be almost absent due to an extremely low conversion of extracellular ATP to ADP by ectoATPase activity [29].

MRS211 and 2-MeSADP are blocker and agonist of P_2Y13_ receptors, respectively.

On the other hand, expression of P2X receptors on rbcs was tested by confocal microscopy, anti-P2X_1-7_ antibodies and isotope ion flux techniques in the absence and presence of subtype specific agonists and antagonists [64,65]. Of these receptors, only P2X_2_ and P2X_7_ were present. Under our experimental conditions only P2X_2_, with a relatively high affinity for ATPe [66–68], would be active. Activation of P2X_7_ is unlikely since: 1- the EC_50_ for ATPe activation is at least three orders higher than the observed ATPe kinetics profile (Fig 1A), and about two orders higher than the EC_50_ for P2X_2_; 2- P2X_7_ activation induces eryptosis that would lead to hemolysis within the time window of the experiment [64,69]. However, MST7 did not induce hemolysis in our study.

Interestingly, the model allows to better understand the observed inhibition of ATPe kinetics by NF110 in terms of the swelling response. Fig 9 shows that, under NF110 exposure, Vr kinetics is inhibited in such a way that—according to the best fit model- the predicted values of Vr remained slightly below Vr^c^, in which case swelling can not affect ATP efflux.

### Effect of ATPe on Vr kinetics

The effect of ATPe on Vr kinetics was monitored under conditions where 1- the accumulated ATPe was removed by apyrase, a nucleotide scavenger; 2- ATP efflux was partially inhibited by CBX or PBC; 3- the effect of ATPe on P receptors was pharmacologically blocked.

As expected CBX, PBC and apyrase significantly inhibited swelling by 40–60% (Fig 2A). However, the fact that 10 U/mL apyrase, thought to fully remove ATPe, caused similar inhibition of swelling as CBX and PBC, which partially inhibit ATP efflux, implied that either part of the swelling did not depend on [ATPe] or apyrase was not efficient enough to rapidly remove the ATP that accumulated at the surface of MST7 activated cells. The latter hypothesis applied, since at higher apyrase concentrations a higher degree of swelling inhibition was observed, with 80 U/mL apyrase completely preventing Vr increase (Fig 2D). Blockage of P2X receptors with NF110 caused an approx. 80% reduction of swelling (Fig 4A).

These results suggest that the whole amplitude of the swelling response depended on ATPe, but only 80% of this amplitude relied on P2X activation by ATPe.

Since P2X receptors act as cation channels with significant Na^+^ conductance [23–25], the observed P2X-dependent Vr increase could be due to activation of net sodium influx, leading to an increase of intracellular osmolarity, followed by uptake of osmotically obliged water. In support of this hypothesis, swelling was almost blunted in assay medium lacking sodium.

Moreover, in agreement with these findings, exposure of rbcs to MST7 induced an acute 60% increase of intracellular sodium (Fig 6).

To mathematically model the effects of ATPe on V_r_ kinetics, we assumed that Vr kinetics can be described by the sum of two exponential functions of time, with a fast phase followed by a slow phase of swelling. Simulations showed that the amplitude of the fast phase depended almost linearly with [ATPe], while the relatively small amplitude of the slow phase remains nearly constant, and was considered independent of P2X activation by [ATPe].

Results of Fig 9 showed a good fit of the model to experimental data in the presence of MST7 alone, MST7+CBX and MST7 + NF110, thus validating the suitability of the model.

### Effect of ectoATPase activity on ATPe kinetics

Results of Fig 12 show the simulated ATPe kinetics resulting from the best fit model, and a comparison to predictions of this kinetics when the magnitude of ectoATPase activity was varied.

We first compared ATPe kinetics in the presence of ectoATPase activity (the complete data-driven model) and in its absence, the latter being a condition where ectoATPase activity is—mathematically- blocked. It can be seen that the difference between both curves was very small. This means that ectoATPase activity, although fully functional in the nanomolar range of physiologically relevant [ATPe] concentrations, does not play a role in shaping ATPe kinetics of rbcs. I.e., under the experimental conditions, the kinetics of [ATPe] mainly reflects the kinetics of ATP release. A very low ectoATPase activity is a unique feature of erythrocytes from human and other mammalian species [70], since in most other cell types from most vertebrate species, ectoATPase activity is much higher and therefore a potential modulator of ATPe kinetics [71,72]. Accordingly, when higher values of ectoATPase activity were fed into the model, the predicted ATPe kinetics departed from the best fitting curve of Fig 10. In erythrocytes high levels of ectoATPase activity occur at least in two different scenarios: 1- under infection by *P*. *falciparum*, ectoATPase activity of rbcs increases about 400 times during the intraerythrocytic cycle of the parasite, thus gaining control of ATPe kinetics [73]; 2- in erythrocytes of *Xenopus*, like in those of many other non-mammalian vertebrate species [70], ectoATPase activity is at least two orders higher than that of rbcs.

### Mutual feedback of Vr and [ATPe]

**Fig 13:**
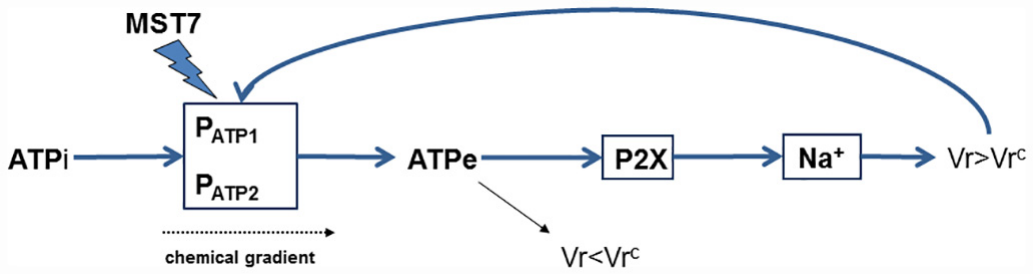
Mutual regulation of ATPe and Vr. The scheme summarizes the key features of this study. In rbcs, exposure to MST7 triggers activation of two ATP permeabilities (P_ATP_^1^ and P_ATP_^2^), causing [ATPe] kinetics. In the absence of P2X activation, swelling is activated by ATPe, but the magnitude of volume increase is so small that it does not trigger ATP efflux. This is shown as the arrow leading to Vr<Vr^c^. Activation of a P2X receptor by ATPe leads to sodium influx, coupled to water influx and swelling. As Vr increases, it surpasses Vr^c^ (i.e., Vr>Vr^c^), so that P_ATP_ is transiently activated, leading to ATP release. ATPi and ATPe denote intracellular and extracellular ATP, respectively. The chemical gradient refers to the difference between ATPi and ATPe at both sides of the plasma membrane.

The scheme of Fig 13 summarizes the key features of this study. Addition of MST7 to viable rbcs induces the activation of two kinetically different ATP permeabilities (Fig 11B), which leads to [ATPe] increase. In the absence of P2X activation, swelling can still be activated by ATPe (consistent with 80 U/mL of apyrase fully blocking cell swelling, (Fig 2D). However, the magnitude of volume increase is so small that it does not trigger ATP efflux. On the contrary, activation of a P2X receptor by ATPe leads to sodium influx, subsequent influx of water and swelling. About 80% of the swelling response is explained by receptor activation. As Vr increases, it surpasses a critical, threshold value Vr^c^ where P_ATP_ can be transiently activated, leading to ATP release. This series of events constitute a positive feedback loop whereby MST7 induces ATP release which then, via P2X→ Na^+^→ swelling, further activates itself.

This loop does not challenge cell survival, because in the post-stimulus phase the swelling response levels off rapidly, so that Vr remains stable at a new level. This is why no hemolysis is detected during MST7 exposure. Moreover, as already mentioned, P_ATP_, which is acutely activated by MST7 and by swelling, is inactivated by time (Fig 1), which explains why [ATPe] level tends to saturate with time. Thus, upon MST7 exposure, even with the acute, approx. 2000 fold increase of P_ATP_ does not impose an energetic burden on cells. In fact, given that intracellular ATP is milimollar, and ATPe kinetics displays in the nanomolar range, the whole ATPe response requires less than 1.3% of the available cytosolic ATP.

### Speculations on the systemic consequences of ATP efflux

The quantitative ATPe profile described in this study for rbcs is compatible with an *in vivo* scenario where, under unstimulated conditions, [ATPe] is maintained stable at a relatively low value [74], and acute increases occur only transiently and in response to certain physiological and/or pathological conditions.

Assuming a 30–40% hematocrit and a linear relationship between [ATPe] and hematocrit, a similar activation of ATP efflux as that of Fig 1 would generate up to 3 μM of erythrocyte-derived ATPe in the vasculature, a concentration suitable for activating most P2 receptors in erythrocytes as well as in endothelial cells [75–77]. Activation of P2Y receptors in endothelial cells induces the generation and release of vasodilators [78,79], followed by relaxation of smooth muscles surrounding the capillaries of the microcirculation and a corresponding relaxation of vascular tone [45].

Erythrocytes are mobile and therefore encounter quite different environments across the circulatory system. As we show in this study, an isosmotic hypotonic challenge, even if minor, can strongly activate ATP efflux. Transient isosmotic swelling of rbcs may occur whenever energy levels are compromised (a consequence of the Gibbs-Donnan equilibrium, [80]), or e.g., when urea is taken up as rbcs leave the renal papilla [81,82]. In certain diseases, altered cation permeability may result in swelling, an example being overhydrated hereditary stomatocytosis [83].

In the future, improved variants of MST7 could be used as pharmacological tools to enhance ATP release from rbcs. This can be particularly important in the treatment of patients with diseases such as hyperinsulinemia, type 2 diabetes and primary pulmonary hypertension among others, where the efflux of ATP in response to various physiological stimuli is highly diminished.

## Supporting Information

S1 File Modeling of ATPe and cell volume in MST7-stimulated rbcs. A data-driven mathematical model was built to account for the dynamic interaction between [ATPe] and relative cell volume (Vr) when rbcs are exposed to MST7. **Fig A. Vr kinetics of MST7-exposed rbcs.** Rbcs were incubated in 200 μL of isosmotic medium (300 mosM) and after 20 min cells were exposed to 10 μM MST7 in isosmotic medium (●) or in hyperosmotic medium (345 mosM; ○). Calibration was performed at the end of each experiment by sequentially exposing rbcs to assay media with the following osmolarities (in mosM)287, 260, 245 or 312, 323 and 340. Results are the mean ± SE of 20–30 rbcs (N = 6) for isosmotic medium and 20–30rbcs (N = 4) for hyperosmotic medium. Numbers of determinations (n) from independent preparations (N) are indicated. **Fig B. Effect of P2Y**_**13**_ **receptor activation on ATPe kinetics.** Prior to exposure to MST7, rbcs were pre-incubated for 10 min with 1 μM 2-MeSADP (dark grey line, N = 3, n = 4) or 1 μM MRS 2211 + 1 μM 2-MeSADP (light grey line, N = 3; n = 4). The dashed arrow indicates addition of treatments and the full arrow indicates exposure to 10 μM MST7. Numbers of determinations (n) from independent preparations (N) are indicated.
(DOCX)
