# Supplementary material for: Dynamic Regulation of Cell Volume and Extracellular ATP of Human Erythrocytes
Source: PLoS One. 2016 Jun 29;11(6):e0158305. doi: 10.1371/journal.pone.0158305 (PMC4927150; doi:10.1371/journal.pone.0158305)
Supplement: S1 File — A data-driven mathematical model was built to account for the dynamic interaction between [ATPe] and relative cell volume (Vr) when rbcs are exposed to MST7. Fig A. Vr kinetics of MST7-exposed rbcs. Rbcs were incubated in 200 μL of isosmotic medium (300 mosM) and after 20 min cells were exposed to 10 μM MST7 in isosmotic medium (●) or in hyperosmotic medium (345 mosM; ○). Calibration was performed at the end of each experiment by sequentially exposing rbcs to assay media with the following osmolarities (in mosM)287, 260, 245 or 312, 323 and 340. Results are the mean ± SE of 20–30 rbcs (N = 6) for isosmotic medium and 20–30rbcs (N = 4) for hyperosmotic medium. Numbers of determinations (n) from independent preparations (N) are indicated. Fig B. Effect of P2Y13 receptor activation on ATPe kinetics. Prior to exposure to MST7, rbcs were pre-incubated for 10 min with 1 μM 2-MeSADP (dark grey line, N = 3, n = 4) or 1 μM MRS 2211 + 1 μM 2-MeSADP (light grey line, N = 3; n = 4). The dashed arrow indicates addition of treatments and the full arrow indicates exposure to 10 μM MST7. Numbers of determinations (n) from independent preparations (N) are indicated. (DOCX) [file pone.0158305.s001.docx]

**Modeling of ATPe and cell volume in MST7-stimulated rbcs**

A data-driven mathematical model was built to account for the dynamic interaction between [ATPe] and relative cell volume (Vr) when rbcs are exposed to MST7. To simplify analysis, we first describe how [ATPe] affects Vr kinetics, and then how Vr affects ATPe kinetics according to the following scheme.

Complete model: Dynamic interaction between V_r_ and [ATPe]

(Eqs. A-M)

Effects of [ATPe] on Vr

(Eqs. A-B)

Effects of V_r_ on [ATPe]

(Eqs. J-M)

**Vr kinetics and its dependence with [ATPe]**

Experimental results show that, in the absence of stimulus, Vr does not significantly change. Exposure to MST7 induced a non-linear Vr increase that was well described by the sum of two increasing exponential functions of time, with a fast phase followed by a slow phase.

Accordingly, a model encoded in the following expression was proposed:

$V_{r}=A_{1}\left( 1-e^{-r_{1}\left( t-t_{s} \right)} \right)+A_{2}\left( 1-e^{-r_{2}\left( t-t_{s} \right)} \right)+1$ $V_{r}=\left\{ \begin{matrix} 1 & t<t_{s} \\ A_{1}\left( 1-e^{-r_{1}\left( t-t_{s} \right)} \right)+A_{2}\left( 1-e^{-r_{2}\left( t-t_{s} \right)} \right)+1 & t \geq t_{s} \end{matrix} \right.$ (A)

Where *t_s_* is the time at which cells were exposed to MST7, and *V*r equals 1 before application of the stimulus, *i.e*., *t*<*t_s_*.

Parameters *r_i_* (*i*= 1, 2) are kinetic coefficients and *A_i_* (*i*= 1, 2) represent the amplitude of the swelling response, with *A_1_* and *A_2_* corresponding to the fast and slow phases, respectively.

Since experimentally Vr is affected by ATPe, either*A_1_*, *A_2_* or both should be a function of [ATPe]. The best fit of Eq. A to experimental data was obtained when *A_2_* is constant, and *A_1_* is a sigmoid function of [ATPe] as follows:

$A_{i}=\frac{A_{i}^{\max}}{1+\left( \frac{\left[ \mathrm{ATPe} \right]_{i}^{50}}{\left[ \mathrm{ATP}_{e} \right]} \right)^{h}}+A_{i}^{0}$ $A_{1}=\frac{A_{1}^{\max}}{1+\left( \frac{\left[ \mathrm{ATP}_{e} \right]_{1}^{50}}{\left[ \mathrm{ATP}_{e} \right]} \right)^{h}}+A_{1}^{0}$ (B)

where *A_1_^max^*, *A_1_^0^* are the maximal and minimal amplitudes respectively. [ATPe]*_1_^50^* is the concentration of ATPe corresponding to the 50% of the amplitude and *h* denotes the degree of sigmoidicity of the amplitude response.

Since all effects of ATPe on *A_1_* are assumed to be caused by P2X activation, the magnitude of *A_1_* will depend on [ATPe] (Eq. B), except under P2X receptor blockage (*i.e*., exposure of cells to NF110), where *A_1_* does not depend on ATPe and equals *A_1_^0^*.

Within the range of ATPe concentrations observed when rbcs are exposed to MST7, *A_1_* is almost a linear function of [ATPe]. The magnitude of d*A_1_*/d[ATPe] represents the sensitivity of *A_1_* to [ATPe].

Modeling of Vr kinetics, and its dependence to [ATPe] (*i.e*., Eqs. A and B), was validated by running a global fit to experimental data in the presence of MST7 alone (control), MST7 + carbenoxolone (denoted as CBX, an inhibitor of pannexin 1 dependent ATP efflux) and MST7 + NF110 (a blocker of P2X_1-3_ receptors). Best fitting curves are shown in Fig. 9, with values of best fit for the parameters given in Table A.

**Modeling ATPe kinetics and its dependence with Vr**

In rbcs, ATPe kinetics triggered by MST7 was modeled considering two contributions: a source given by a non-lytic release of intracellular ATP (*J_ATP_*), which increases [ATPe], and a sink due to the rate of ATPe hydrolysis by ectoATPase activity (*J_ATPase_*), which decreases [ATPe]:

$\frac{d\left[ {ATP}_{e} \right]}{dt}=J_{ATP}-J_{ATPase}$ (C)

**The rate of ATP consumption (*J_ATPase_*)**

We have shown before [1] that, within the range of [ATPe] observed in Fig. 1, the experimental ectoATPase activity of intact viable rbcs follows a linear function with [ATPe], and is not affected by changes in Vr.

Thus, J_ATPase_ was expressed as:

$J_{ATPase}=k_{ATP}\left[ {ATP}_{e} \right]$ (D)

Where k_ATP_ is the first order constant of ectoATPase activity [1].

**Non-lytic release of ATP (*J_ATP_*)**

ATP release occurs in the absence of lysis, and is mediated by an unsaturated facilitated diffusion mechanism as:

$J_{ATP}=P_{ATP}\left( \left[ {ATP}_{i} \right]-\left[ {ATP}_{e} \right] \right)$ (E)

Where P_ATP_ denotes a permeability factor whose properties are explained below, and [ATPi] and [ATPe] are the intracellular and extracellular ATP concentrations, respectively. The difference between [ATPi] and [ATPe] is proportional to the chemical gradient of ATP across the plasma membrane.

Since the experimentally observed changes in [ATPe] occur in the nanomolar range (Fig. 1) whereas [ATPi] is millimolar [1], Eq. E can be approximated to:

$J_{ATP}\sim P_{ATP}\left[ {ATP}_{i} \right]$ (F)

Exposure to MST7 did not significantly affect the intracellular mass of ATP [1]. Still, [ATPi] can in principle vary as a consequence of Vr change. However, we showed previously that the calculated decrease in [ATPi] induced by MST7 dependent swelling was very small, and therefore did not significantly affect *J_ATP_* [1]. Thus, [ATPi] was considered constant in Eq. F.

Considering Eqs. D and F, ATP kinetics modeled by Eq. C can now be written as:

$\frac{d\left[ {ATP}_{e} \right]}{dt}=P_{ATP}\left[ {ATP}_{i} \right]-k_{ATP}\left[ {ATP}_{e} \right]$ (G)

**Properties of P_ATP_**

Since inhibition of pannexin1 by CBX did not fully abolish ATP efflux (Fig. 2 B), the existence of two conduits mediating ATP release was assumed in the model, so that:

$P_{ATP}=P_{ATP}^{1}+P_{ATP}^{2}$ (H)

Where P_ATP_*^2^* accounts for ATP transport by pannexin 1, while P_ATP_*^1^* corresponds to the permeability of a yet unidentified ATP conduit.

Time dependent changes in P_ATP_ are brought about by different degrees of activation of the two conduits responsible for ATP efflux (Eq. I, see below). That is, in the unstimulated condition there is a basal stationary state (o) of P_ATP_; exposure to MST7 induces an instantaneous irreversible transition to a stimulated state (s), followed by a subsequent irreversible decay to a final state (f).

Accordingly, time dependent values of P_ATP_*^1^* and P_ATP_*^2^* are given by the functions *P_1_*(*t*) and *P_2_*(*t*):

$P_{i}\left( t \right)=\left\{ \begin{matrix} \frac{p_{0}}{2} & t<t_{s} \\ p_{f}^{i}+e^{-k_{i}(t-t_{s})}\cdot\left( p_{s}^{i}-p_{f}^{i} \right) & t\geq t_{s} \end{matrix} \right.$ (I)

Where *p_0_* represents the total ATP permeability of both conduits before the stimulus is applied, while *p_s_^i^* and *p_f_^i^* (*i=1, 2*) characterize the stimulated and final states for each of the two ATP conduits, respectively.

Experimental results of Fig. 3 showed that swelling affects ATP release. This feature was included in the model by weighting each ATP conduit permeability *P_i_*(*t*) by a function of *V_r_*. This weight was modeled as a step function *H* as follows:

$H\left( V_{r} \right)=\left\{ \begin{matrix} h^{0} & V_{r}<V_{r}^{c} \\ h^{f} & V_{r}\geq V_{r}^{c} \end{matrix} \right.$ (J)

When *Vr* overcomes the critical value Vr^c^, H (Vr) switches from *h_0_* to *h_f_*, mimicking a sudden activation of P_ATP_.

In principle, the effects of swelling and time on one or both ATP conduits can be expressed as:

$P_{ATP}\left( t,V\left( t \right) \right)= P_{ATP}^{1}\left( t,V\left( t \right) \right)+P_{ATP}^{2}\left( t \right)=P_{1}\left( t \right) H\left( V \right)+P_{2}\left( t \right)$ (K)

$P_{ATP}\left( t,V\left( t \right) \right)= P_{ATP}^{1}\left( t \right)+P_{ATP}^{2}\left( t,V\left( t \right) \right)= P_{1}\left( t \right)+P_{2}\left( t \right) H\left( V \right)$ (L)

$P_{ATP}\left( t,V\left( t \right) \right)= P_{ATP}^{1}\left( t,V\left( t \right) \right)+P_{ATP}^{2}\left( t,V\left( t \right) \right)=\left( P_{1}\left( t \right)+P_{2}\left( t \right) \right) H\left( V \right)$ (M)

However, from a mathematical perspective, Eqs. K and L are indistinguishable, thus only Eqs. L and M were used to account for Vr affecting one conduit or both conduits, respectively.

Summarizing the effects of Vr on ATPe kinetics, Vr kinetics is given by Eqs. A-B, and the resulting values of Vr at any time are fed into the ATPe kinetics given by Eqs. G-M.

Table A. Parameters of the model.

| **Kinetics of cell volume (Vr)** | | | | | |
| --- | --- | --- | --- | --- | --- |
| **Parameters of Equation B** | | | | | |
| **First exponential term** | | | | | |
| *A_1_^0^* | | (3 ±1).10^-5^ | | | |
| *A_1_^max^* | | 0.435 ± 0.003 | | | |
| [*ATPe*]^50^ (nM) | | 694.03 ± 0.01 | | | |
| *h* | | 1.39 ± 0.04 | | | |
| *r_1_* (10^-2^ s^-1^) | | 7.2 ± 0.7 | | | |
| *dA_1_/d*[ATPe] (1/nM) | | (3.70 ± 0.06).10^-4^ | | | |
| **Second exponential term** | | | | | |
| *A_2_* | | (1.65 ± 0.02).10^-2^ | | | |
| *r_2_* (10^-2^ s^-1^) | | 4.3 ± 0.7 | | | |
| **Kinetics of [ATPe]** | | | | | |
| **ATP permeability (P_ATP_). Equation I** | | | | | |
|  | **MST7** | | **MST7 + NF110** | **MST7 + Hyper** | **MST7 + CBX** |
| *p_0_* (10^-9^ s^-1^) | 0.9 ± 0.2 | | 0.8 ± 0.2 | 0.8 ± 0.2 | 0.8 ± 0.4 |
| *p_s_^1^* (10^-6^ s^-1^) | 3 ± 1 | | | | 0.7 ± 0.1 |
| *p_s_^2^* (10^-6^ s^-1^) | 0.30 ± 0.01 | | | | -- |
| *p_f_^1^* (10^-8^ s^-1^) | 0.90 ± 0.01 | | | | 0.7 ± 0.1 |
| *p_f_^2^* (10^-8^ s^-1^) | 0.081 ± 0.001 | | | | -- |
| *k_1_* (10^-2^ s^-1^) | 5.8 ± 0.6 | | | | 1.438 ± 0.001 |
| *k_2_* (10^-2^ s^-1^) | 0.73 ± 0.04 | | | | -- |
| **Step function H(Vr). Equation J** | | | | | |
| *h_0_* | 1.090 ± 0.005 | | | | |
| *h_f_* | 0.488 ± 0.005 | | | | |
| Vr*^c^* | 1.018 ± 0.001 | | | | |
| **EctoATPase activity (J_ATPase_). Equation D** | | | | | |
| *k_ATP_* (10^-5^ s^-1^) | 1.98 | | | | |

Results are values of best fit obtained by fitting the model of Eqs. A-M to experimental data.

J_ATPase_= ectoATPase activity; k_ATP_ is the first order constant of ectoATPase activity, taken from Leal Denis et al. 2013.

Hyper: hyperosmotic medium

MST7= mastoparan 7, CBX= carbenoxolone

*r_i_*(*i*= 1, 2) = kinetic coefficients

*A_i_* (*i*= 1, 2)= amplitude of the swelling response

*p_0_* = total ATP permeability of both conduits before the stimulus is applied

k(1,2)= rate constants of the exponential functions describing P_ATP_(t).

*p_s_^i^* and *p_f_^i^*(*i=1, 2*) = stimulated and final states for the permeabilities of the two ATP conduits.

h(o,f)= initial and final states of the step function H.

*V_r_^c^*=critical relative cell Volume

**References**

1. Leal Denis MF, Incicco JJ, Espelt MV, Verstraeten S V., Pignataro OP, Lazarowski ER, et al. Kinetics of extracellular ATP in mastoparan 7-activated human erythrocytes. Biochim Biophys Acta - Gen Subj. 2013;1830(10):4692–707.

Fig. A





Fig. B
